# Toward uncertainty-aware manual delineation of brain tumours using eye-tracking and image-derived features

**DOI:** 10.1016/j.phro.2026.101056

**Published:** 2026-08-04

**Authors:** Nina Baumgartner, Daria Laslo, Laura Fontanesi, Catherine R. Jutzeler, Arzu Çöltekin, Sarah Brüningk

**Affiliations:** a ETH Zurich, Rämistrasse101, 8092 Zürich, Switzerland; b Swiss Institute of Bioinformatics, Lagerstrasse 5, Lausanne, Switzerland; c Fachhochschule Nordwestschweiz FHNW, Hochschule für Informatik, 5210 Windisch, Switzerland; d Department of Radiation Oncology, Inselspital, Bern University Hospital and University of Bern, Switzerland; e Department of Digital Medicine, University of Bern, Bern, Switzerland

**Keywords:** Tumour segmentation, Segmentation uncertainty, Eye-tracking, Mouse-tracking, Paediatric brain tumour

## Abstract

**Background and Purpose:** Accurate tumour delineation is key in radiotherapy workflows. Concurrently, diffuse tumour types are inherently difficult to delineate, rendering contour uncertainty information highly valuable for downstream treatment planning. In this study, we investigated whether uncertainty in manually delineated tumour contours can be inferred from clinician behaviour (eye and mouse movements) and image-derived indicators.

**Materials and Methods:** In a two-stage controlled experiment, 36 clinical imaging experts described and manually delineated brain tumours on T2-FLAIR (fluid-attenuated inversion recovery) MRI scans, while their eye and mouse movements were recorded. Inter-observer contour variability was used as a proxy for contour uncertainty. In addition, we extracted image-derived features, including U-Net saliency maps, pixel-wise U-Net segmentation probabilities, and image entropy. To assess and predict contour uncertainty, we applied mixed-effects models and machine learning algorithms.

**Results:** Higher contour uncertainty was associated with higher saccade velocities and greater fixation density. Uncertainty was also significantly correlated with segmentation error and image-derived features (*p* < 0.05). A random forest regressor, combining behavioural and image-derived features, explained 39% of the variance in contour uncertainty. Notably, features derived from downsampling layers were the strongest predictors, despite displaying the lowest saliency overlap with human attention.

**Conclusions:** Our results suggest that uncertainty in manually delineated tumour contours can be estimated using behavioural and image-derived features, without explicit uncertainty annotations. While clinical deployment will require validation under realistic acquisition conditions and with specialty experts, these findings established the feasibility of passively inferred uncertainty as a foundation for future uncertainty-aware delineation tools.

## Highlights

• Gaze patterns differed by task (p < 0.001) and distinguished high-performing raters.
• Combined behavioural and image features explained 39% of segmentation uncertainty.
• Random forest predicted uncertainty for unseen scans and observers (R^2^ = 0.4, MAE = 0.2).

## Graphical abstract

**Figure:**
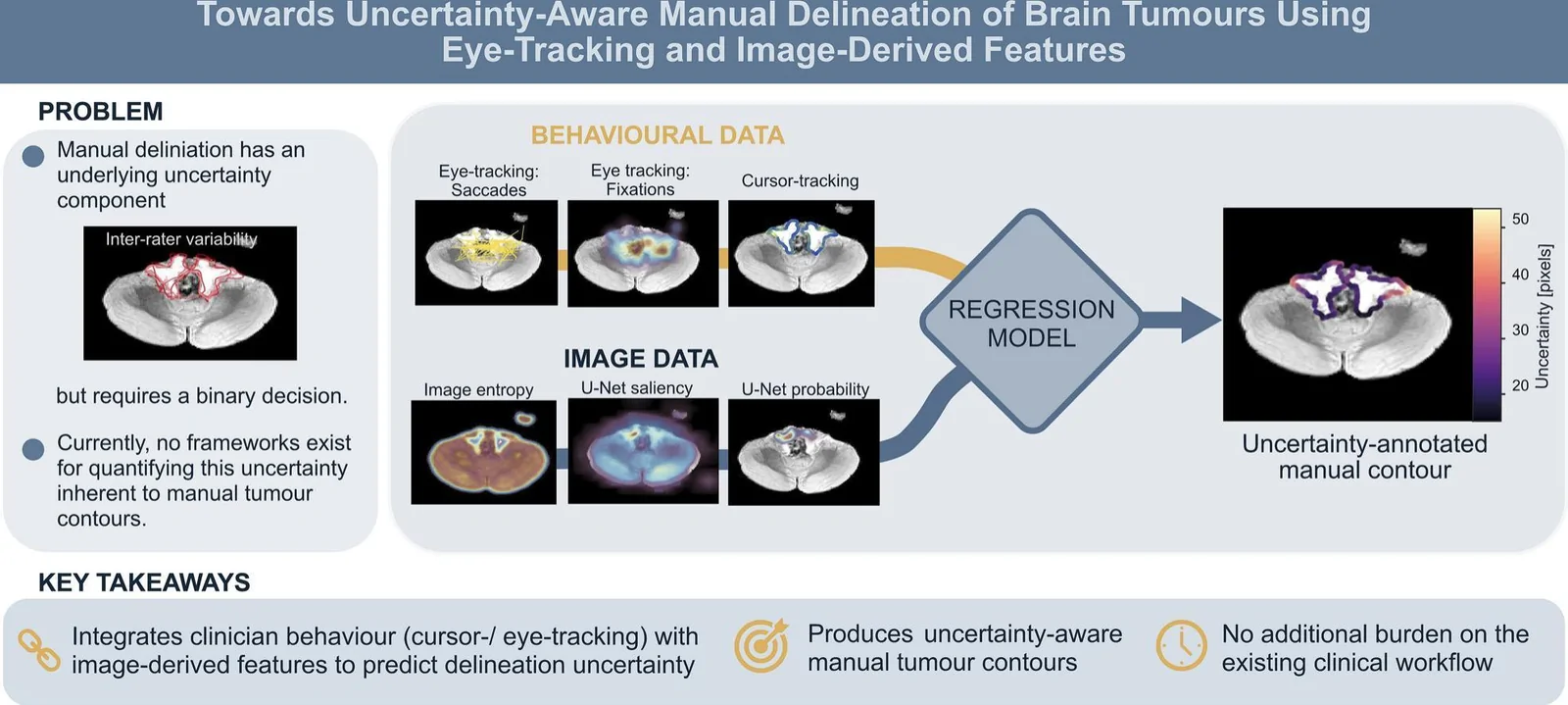

## 1 Introduction

Tumour segmentations are critical for radiotherapy planning and response assessments [1], [2], but may vary substantially within and between participants, particularly for diffuse tumours [3], [4]. Paediatric diffuse midline gliomas (DMGs) [5], [6], are a prime example of such a phenotype. Their diffuse and infiltrative growth, atypical contrast enhancement patterns, and heterogeneous subregions make delineation particularly difficult [7], [8], [9].

Despite inherent tumour contour ambiguity, segmentation enforces a binary decision. This information loss contributes to increased contour variability, with downstream impacts on treatment planning, and outcome evaluation [10]. This is particularly important for radiotherapy, where dose distributions are derived based on segmentations [11], [12]. Despite the recent emergence of probabilistic volume definitions [13], [14], binary contours remain the standard. While uncertainty quantification [15], [16], [17], [18] is critical to enhance interpretability, robustness, and clinical relevance of automated tools [19], no comparable frameworks exist for manual segmentations.

In a preliminary survey of 18 medical professionals in Switzerland (Supplementary A, Fig. S1), respondents highlighted such contour uncertainty annotations as useful (median 8/10), specifically for personalised *radiotherapy planning* and simplified *communication with colleagues*. Many reported that uncertainty on manual contours could increase their trust in colleagues (50%) and auto-segmentations (61%), provided standardized definitions were available, and minimal additional workload. Despite a growing recognition that contours carry uncertainty, no method currently exists to estimate contour uncertainty without explicit annotation, limiting quality assurance and downstream integration.

Eye tracking is a non-intrusive method to measure visual attention [20], [21] within clinical decision-making processes [22], [23]. Patterns of gaze were previously linked to strategies in medical image perception [24]. Gaze dynamics, comprising fixations (periods of relatively stable gaze on a specific location) and saccades (rapid eye movements between fixation points), could indicate uncertainty in object detection [23], [24].

We therefore aimed to investigate whether manual contour uncertainty is inferable from the clinicians' eye and mouse movements combined with image-derived features. We proposed a framework that integrates behavioural and image-derived signals to estimate contour uncertainty, examining their association across participants and cases, as groundwork for uncertainty annotations in manual segmentation. As proof of concept, we focused on DMGs and presented data collected across international expert annotators at the 2025 European Conference of Radiology (ECR2025).

## 2 Materials and methods

### 2.1 Stimuli and materials

Visual stimuli comprised 12 2D axial slices from the MRIs of ten DMG patients from the paediatric 2023 Brain tumour Segmentation (BraTS) Challenge [25], [26]. Ground truth tumour segmentation masks, provided by volunteer neuroradiologists and reviewed by board-certified neuroradiologists, were available (Supplementary B). Patients were randomly selected, and automated segmentations were generated using a U-Net-based model [27]. Following DMG clinical guidelines, we focused on T2-weighted fluid-attenuated inversion recovery (T2-FLAIR) images [28]. The scans were randomly distributed into three sets of balanced tumour size, axial slice position, and image entropy (Table S1, S2). For nine patients, the central axial tumour slice was selected. The remaining patient was classified more difficult to segment based on our computational criterion (U-Net Dice <0.81) and three equally spaced slices were extracted, one per group. The segmentation model is a 2D U-Net trained on paediatric BraTS2023 T2-FLAIR slices [25], [26] (Supplementary C).

For eye- and mouse- tracking, we used the EyeLink Portable Duo system (SR Research) and WebLink software (v2.2.161.0, SR Research). The system was calibrated for each user independently. Scans were presented on a 520 × 325 mm screen at ∼550 mm.

### 2.2 Experimental procedure

We recruited 36 participants at ECR2025 (all radiology professionals with completed medical education) according to the ethical approval waiver (Bernese Ethics Commission). They were assigned to one of the three experimental groups in sequential order, completed a demographics questionnaire (age, area of expertise, country of work, eye conditions, sex, segmentation and clinical experience), and provided participation consent. Following system calibration and validation (using two randomised five-point sequences), each participant analysed four scans across two 1-min tasks: (1) verbally describe the tumour boundaries and (2) delineate the tumour using the mouse cursor. During delineation, the mouse cursor was visible but no mouse trace was shown. Eye- and mouse-tracking data were continuously recorded (Supplementary D, Fig. S2).

### 2.3 Data processing

Data was extracted using EyeLink's DataViewer [29]. Fixations, saccades, and blinks were detected via EyeLink's On-Line Parser [30], using conservative thresholds (40°/s velocity, 8000°/s^2^ acceleration, ≥ 0.2° motion for saccades; ≥ 12 ms for blinks). A fast velocity filter and pursuit velocity cap (60°/s) were applied. Post-hoc drift correction was performed for four participants. Fixations under 80 ms, outside-scan events, saccades overlapping blinks, and trials (unique participant-scan combinations) with short fixation times (<3 s) were excluded. Mouse tracks were manually cropped and connected, generating tumour contours and binary segmentation masks. When multiple contours were available, only the final complete loop was retained.

The generated masks were compared to the ground truth segmentation masks by Dice similarity coefficient and Hausdorff distance [31]. Participants who failed to identify the tumour in any trial (Dice = 0, *n* = 7) were excluded. The remaining participants were stratified into: “high-performers” (*n* = 14, top 50% on both metrics in at least 2 scans) and “low-performers” (n = 14).

Behavioural metrics (including saccade amplitude and velocity, fixation duration and precision, mouse velocity, task time, fixation/saccade counts, scan path length, and fixation dispersion and entropy) were computed for each participant and task (Table S3, S4). Fixation maps were generated using a Gaussian kernel weighted by fixation duration, normalised to [0,1], and binarized (Otsu's method). Task- and performance-related differences across demographic variables, behavioural and image-derived features, were assessed using rank biserial correlation, Mann-Whitney U, Wilcoxon signed-rank tests, and visual comparison of averaged gaze maps.

Image-derived features were computed for each scan to capture local intensity heterogeneity and spatial patterns of U-Net saliency. Matching the input available to human observers, the U-Net segmentation model (Supplementary C) was used to generate raw sigmoid outputs (probability) and Grad-CAM–based saliency maps [32], [33] (Supplementary C). Local image complexity was quantified using Shannon entropy (4-pixel radius), and all 240 × 240 feature maps were upsampled (nearest-neighbor) to the 1488 × 1108 (resolution in experiment).

### 2.4 Uncertainty quantification

**Fig. 1:**
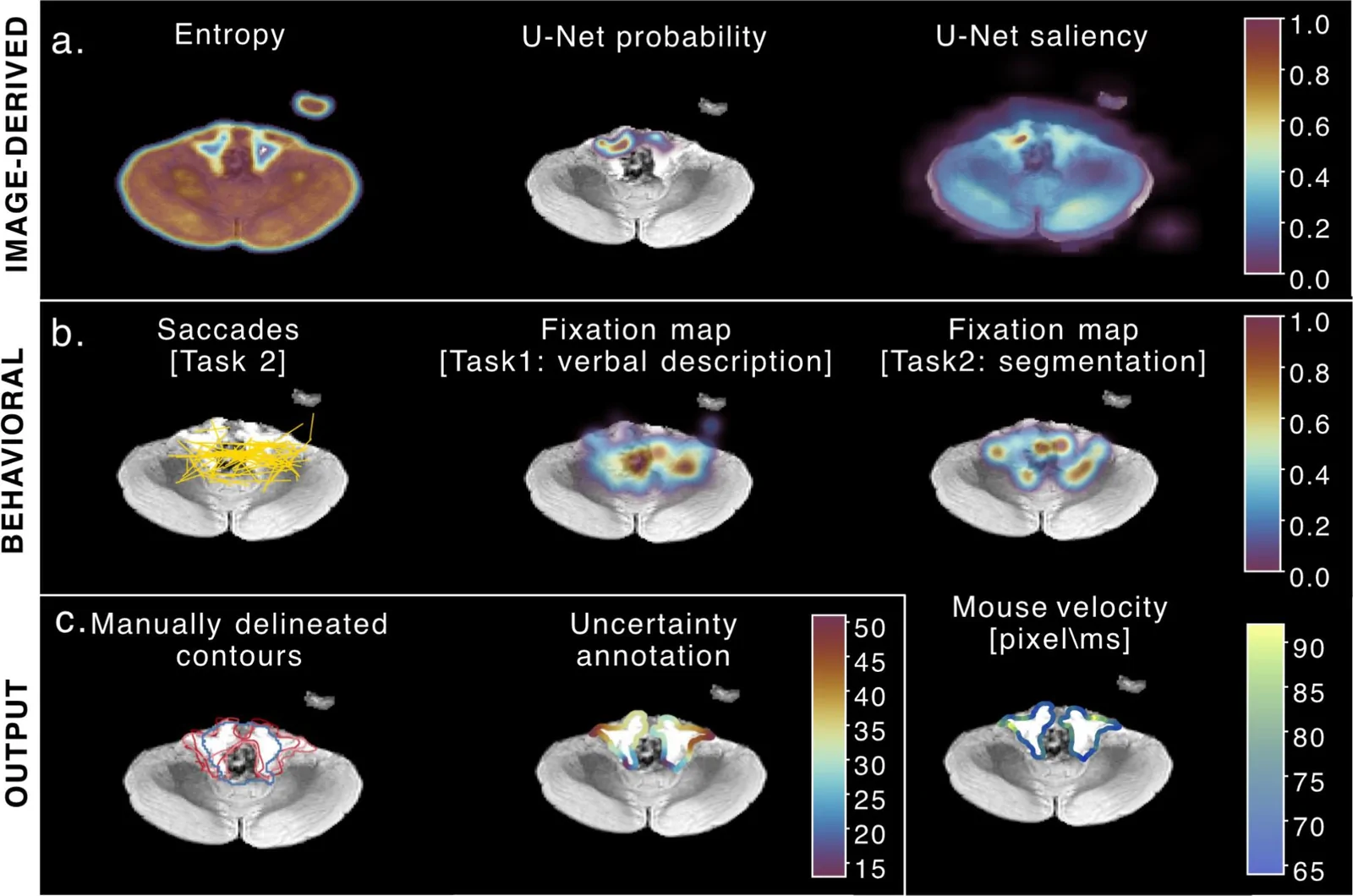
Annotations overview for a sample trial. (a) Image-derived annotations: pixel-wise entropy, U-Net probability, and U-Net saliency maps (all normalised [0,1]). (b) Behavioural annotations: saccades, fixation heatmap (Task 1), fixation heatmap (Task 2), and mouse velocity [pixels/ms] (Task 2). (c) tumour contours: manually delineated tumour contours from three randomly selected participants (red) with overlaid ground truth (blue); uncertainty-annotated contour for the selected sample (variability quantified as the average minimal distance to all other outlines). (For interpretation of the references to colour in this figure legend, the reader is referred to the web version of this article.)

Spatial inter-participant variability, considered as proxy for segmentation uncertainty [3], [34], was defined for each contour point x of a participant (p) as the mean, across remaining participants q, of the minimum Euclidean distance from x to each of their outlines (Eq. 1), where Pis the set of participants, $C_{q}$ is the manually delineated contour of participant q and y is a point on that contour over which the minimum distance is taken (Eq. 1). For each point, local data were aggregated within a 20-pixel radius, chosen based on eye-tracker accuracy [35] and human visual acuity [36] (Fig. S3). For every contour point, (1) eye-tracking, (2) mouse-tracking, and (3) image-derived features were computed alongside the uncertainty value (Fig. 1). Missing fixation values were zero-imputed, missing saccade values were replaced using the nearest saccades, behavioural features were min-max normalised within participants, and uncertainty was log-transformed.

(1) $$u\left(x\right)=\frac{1}{|P|-1}\;\sum_{p\neq q}\min_{y\in C_{q}}\;{\left\|x-y\right\|}_{2}\;$$

To identify associations with segmentation uncertainty, linear mixed-effects models were fitted to behavioural and image-derived features, with random intercepts for participants and scans. Performance was assessed via marginal and conditional $R^{2}$ and key predictors were identified through variable importance and significance testing (α<0.05). Additionally, two models (variance inflation factor and random forest), accounting for the multicollinearity of the variables (Fig. S4), were trained.

### 2.5 Segmentation uncertainty prediction

Random forest models were trained to predict the log-transformed uncertainty values at each contour point from behavioural and image-derived features using nested cross-validation, with outer leave-one-group-out splits and inner leave-one-scan-out validation for hyperparameter tuning (Supplementary E). Classifiers were also trained on binarized uncertainty values (Supplementary F).

### 2.6 Differences between human attention and U-net saliency

Human attention maps were generated by averaging fixation maps across all participants for each scan and task. U-Net saliency maps from all convolutional layers and the human attention maps were binarized using Otsu thresholding [37]. For each scan, the overlap between human attention and U-Net saliency across all task-layer combinations was quantified with Dice and cosine similarity. Model uncertainty, estimated via test-time augmentation, showed no consistent trial-level association with human uncertainty and was not used as a predictor (Fig. S5). All code used for the preprocessing and analysis in this study is available at https://gitlab.ethz.ch/BMDSlab/publications/oncology/manual-segmentation-uncertainty-using-eyetracking.

## 3 Results

Thirty-six medical professionals participated in the experiment. Each participant rated four scans, resulting in a total of 144 unique trials. Following preprocessing, 34 trials were excluded due to data quality issues or failure to identify the tumour (Dice = 0, Fig. S6, S7). Participants were evenly distributed across experimental groups with comparable demographic and segmentation experience (Table 1). The final dataset comprises 110 trials from 28 participants with each scan annotated by 6–10 participants.

**Table 1:** Participant demographic information and segmentation expertise summary by experimental group and overall. Segmentation expertise was self-rated on a scale from 1 (‘Never done a segmentation’) to 5 (‘Performed hundreds of segmentations’). Years of experience were reported starting at the beginning of residency. Values are reported as mean ± standard deviation.

Metric	Group 1 (*n* = 11)	Group 2 (*n* = 10)	Group 3 (*n* = 7)	Overall (*n* = 28)
**Age [years]**	35.3 ± 7.3	31.9 ± 6.1	38.6 ± 10.1	34.9 ± 7.8
**Segmentation Expertise**	2.1 ± 1.4	2.2 ± 1.3	2.3 ± 1.5	2.2 ± 1.4
**Level of Experience [years]**	8.5 ± 5.5	5.5 ± 4.0	11.4 ± 9.5	8.1 ± 6.5
**Sex Female (%)**	54.5	30.0	42.9	42.9
**Sex Male (%)**	45.5	70.0	57.1	57.1

**Fig. 2:**
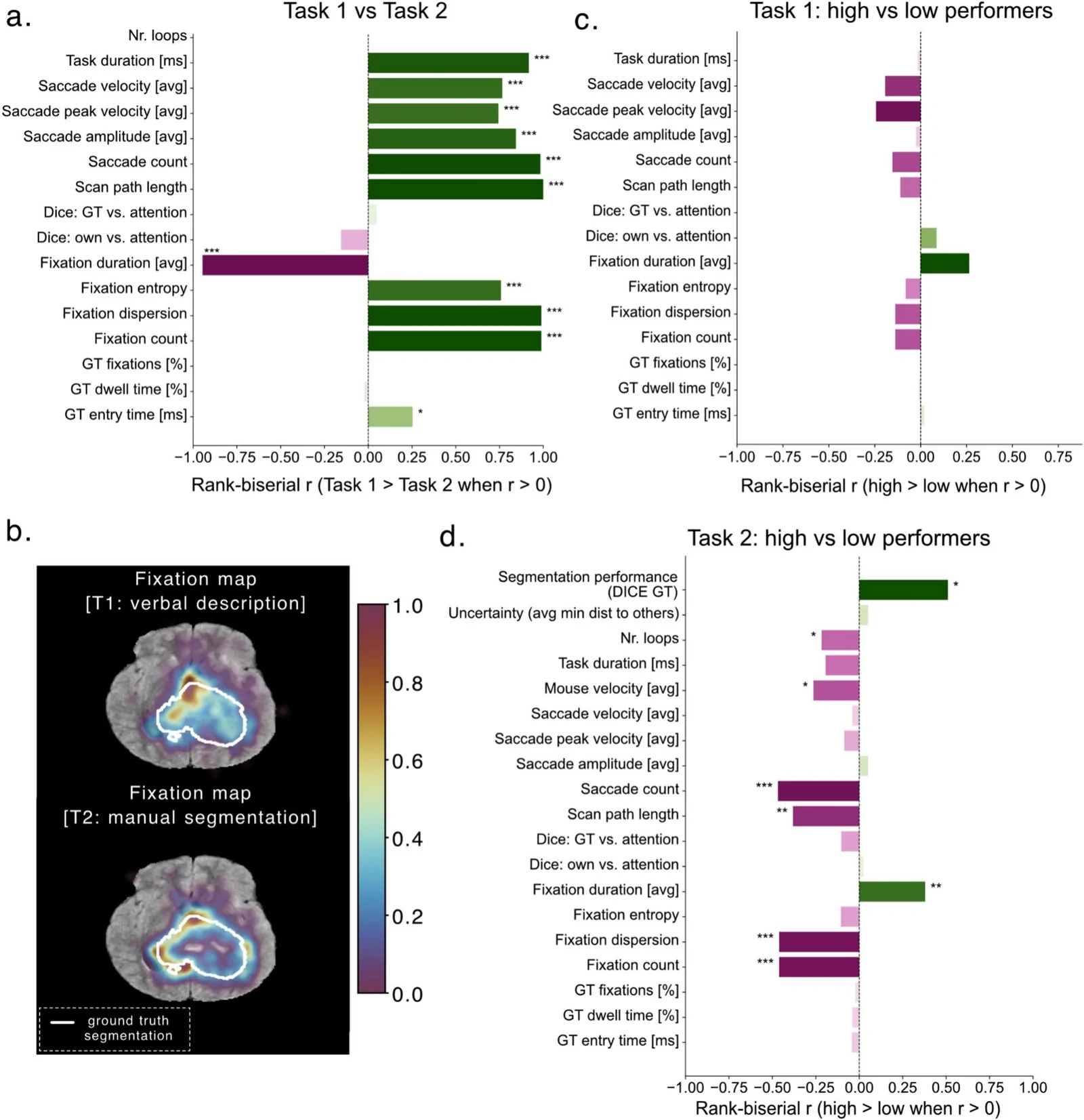
Analysis of human attention in the verbal description and manual segmentation tasks. (a) Differences in eye-tracking metrics between the exploratory task (Task 1) and the segmentation task (Task 2), quantified using rank biserial correlation. Positive values indicate higher metric values in Task 1; negative values indicate higher values in Task 2. (b) Task 1 and Task 2 participant-averaged attention map for a representative scan overlaid with ground truth tumour segmentation (white). (c) Rank biserial correlation for differences in Task 1 and general trial metrics between the performance groups. Positive values indicate higher metric values in high-performers; negative values indicate higher values in low-performers. (d) Rank biserial correlation for differences in Task 2 metrics between the performance groups. Positive values indicate higher metric values in high-performers; negative values indicate higher values in low-performers. (a,c-d) Shown is a selection of variables; the full description of the variable set is available in Supplementary Table S4. Asterisks denote statistical significance from Mann-Whitney *U* Test comparing value distributions across the two tasks (*** *p* < 0.001, ** *p* < 0.01). The Dice score was calculated between the binarized attention maps and the participants' own as well as the ground truth (GT) segmentation masks.

Task 1 (verbal description) and Task 2 (manual segmentation) showed markedly different gaze patterns (*p* < 0.001). Participants spent longer on Task 1 (+19.6 s) and showed more exploratory viewing: more fixations and saccades, longer scan paths, higher dispersion and entropy, and faster, larger saccades. Task 2 was shorter and characterized by longer fixations (+231 ms) (Fig. 2a). Task 1 fixations were broadly distributed, whereas Task 2 fixations concentrated around tumour boundaries (Fig. 2b).

Beyond task-based differences, visual behaviours were significantly correlated for some individuals across tasks (Fig. S4). Participants who detected the tumour earlier tended to exhibit less exploratory behaviour during annotation, as reflected by shorter scan paths and fewer fixations and saccades.

Scan-specific features (tumour size, axial position, image entropy) or participant characteristics (age, experience, segmentation expertise) were not associated with segmentation performance. In contrast, U-Net Dice scores significantly correlated with human performance (*|r|* = 0.4, *p* < 0.001). The mixed-effects analysis confirmed that for every 0.1 increase in the U-Net Dice score, participant performance improved by an estimated 0.03 ($\beta_{1}=0.3$, *p* < 0.001, Table S5). Notably, even when the algorithm failed (Dice = 0), participants were still expected to segment 50% of the tumour ($\beta_{0}$ = 0.5, 95% CI: [0.4, 0.6]).

High-performers and low-performers did not differ significantly in demographics or self-reported expertise (Supplementary G). However, high-performers displayed distinct visual behaviours (Fig. 2c,d): slower average and peak saccade velocities and longer fixations in Task 1. In Task 2, high-performers demonstrated a more efficient assessment with fewer loops (1 less), fewer fixations and saccades (−8, −9), shorter scan paths (−462.8 pixels) and more spatially focused attention (lower fixation dispersion), decreased mouse velocity, longer fixations, shorter saccades, and faster peak saccade velocities. Task 1 attention centred on the tumour core, while Task 2 attention tracked boundaries, a pattern more pronounced in high-performers (Fig. S8).

A total of 20,238 points from the manually delineated contours across 110 trials were annotated with eye-tracking, mouse-tracking, and image-derived features (Table S6, S7, Fig. S9). Notably, segmentation error correlated with segmentation uncertainty (*|r|* = 0.6), while image-derived features such as summed U-Net saliency and output-layer saliency negatively correlated (*|r|* = 0.3 and 0.4), i.e. less uncertainty in regions of higher U-Net saliency. No consistent difference in uncertainty between performance groups was observed (*p* = 0.52).

To investigate factors associated with segmentation uncertainty, we fitted mixed-effects models evaluating behavioural and image-derived features both jointly and separately. The combined model (Table S8) yielded the best overall fit (marginal R^2^ = 0.30). By comparison, behavioural-only models explained little uncertainty via fixed effects (marginal R^2^ = 0.02; conditional R^2^ = 0.50), while image-derived models demonstrated higher explanatory power (marginal R^2^ = 0.24; conditional R^2^ = 0.62), highlighting the dominant role of image-derived features alongside the considerable participant- and scan-specific contributions. The strongest model predictors were image-derived (Table 2). Increased uncertainty was associated with higher U-Net saliency in the output and encoder layers $\left(p<0.001\right)$, whereas greater U-Net saliency in upsampling layers was associated with lower uncertainty. Although weaker, behavioural signals added explanatory value when integrated. Specifically, lower uncertainty was associated with longer fixation durations during the verbal description task (β = −0.2, p<0.001), whereas increased uncertainty was predicted by higher average saccade velocities (β = 0.3, p<0.001) and greater fixation density during segmentation relative to the descriptive task (β = 0.1, p=0.0198). The variable importances in the mixed-effects model with prior feature selection to address multicollinearity were not considerably different (Table S8).

**Table 2:** Fixed effects from the combined linear mixed model predicting log-variability. Shown are the top six strongest contributing factors (highest “Estimate” values). For the complete model, see Supplementary Table S8.

Predictor	Estimate	Std. Error	df	t value	Pr(>|t|)
(Intercept)	1.3	0.1	22.9	20.9	<2e^−16^
GradCAM out convolutional layer [avg]	9.6	1.1	20,220.0	8.4	<2 × 10^−16^
GradCAM up-layer 1 [avg]	−7.5	1.1	20,230.0	−6.7	2.2 × 10^−11^
GradCAM up-layer 3 [avg]	−1.0	0.1	20,230.0	−12.5	<2 × 10^−16^
GradCAM sum of all layers [avg]	−1.0	0.1	20,080.0	−11.9	<2 × 10^−16^
GradCAM down-layer 2 [avg]	0.5	0.01	20,190.0	33.8	<2 × 10^−16^
Task 2 average saccade velocity [avg]	0.3	0.05	18,570.0	6.8	1.2 × 10^−11^

**Fig. 3:**
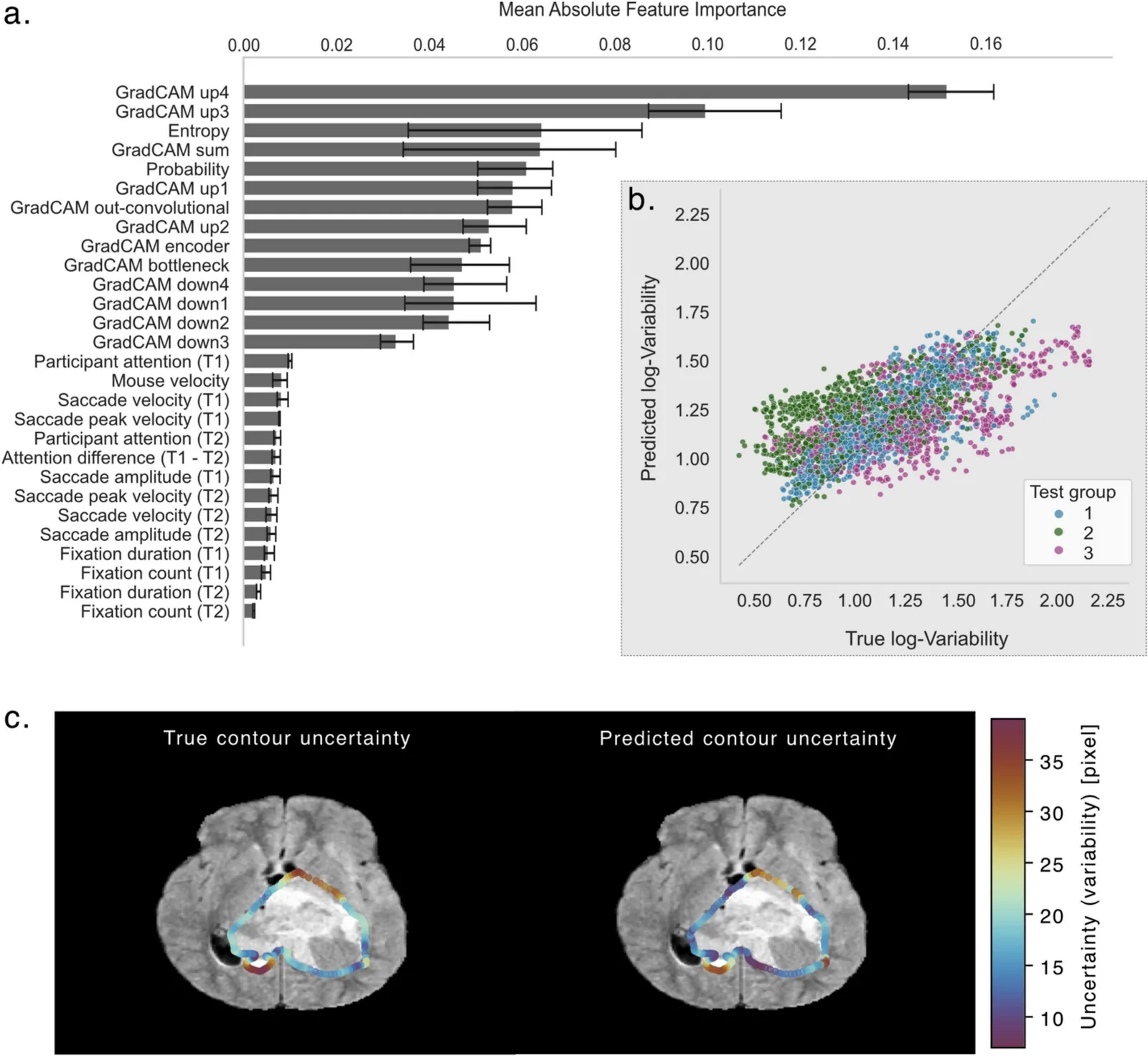
Overview of regression model results based on combined eye-tracking, mouse-tracking, and image-derived features. (a) Feature importance: Mean feature importance (model weights) for each predictor in the model, aggregated across test folds. Error bars indicate the range (minimum to maximum) across folds. T1 = Task 1; T2 = Task 2). (b) Regression performance: Predicted versus true values across all test groups, illustrating model accuracy. A random 15% subset of values is shown for clarity. Mean Absolute Error (MAE) is reported per fold; overall MAE = 0.2. (c) Visualization of true values and predicted uncertainty values displayed in pixels. The description of all variables is available in Supplementary Table S6.

Random forest regression was used to predict uncertainty, confirming the importance of image-derived features, as reflected by higher model weights (Fig. 3a, b). The final model explained 39.2% of the variance in uncertainty across all testing groups ($R^{2}$=0.4), with a MAE of 0.2 (0.1–0.2, Fig. 3c), corresponding to 60.0% of the true SD (Table S6). The top predictors were scan-derived features, particularly U-Net saliency from the third and fourth upsampling layers and image entropy (Fig. 3a): higher entropy and stronger decoder saliency were associated with higher uncertainty, whereas higher U-Net probabilities (indicating stronger model classification of the pixel as tumour) and encoder saliency predicted lower uncertainty. Among the behavioural features, faster mouse movements and higher saccade velocities were most indicative of increased uncertainty (Fig. S10).

Despite variability in MAE/SD across individual participants (0.2–1.1) and scans (0.6–1.8; Fig. S11), the model demonstrated an overall strong performance on unseen participants and scans. Model performance was unrelated to participant age, experience, and self-rated segmentation expertise; however, it was higher for participants with minimal segmentation uncertainty and superior segmentation performance (Fig. S12).

**Fig. 4:**
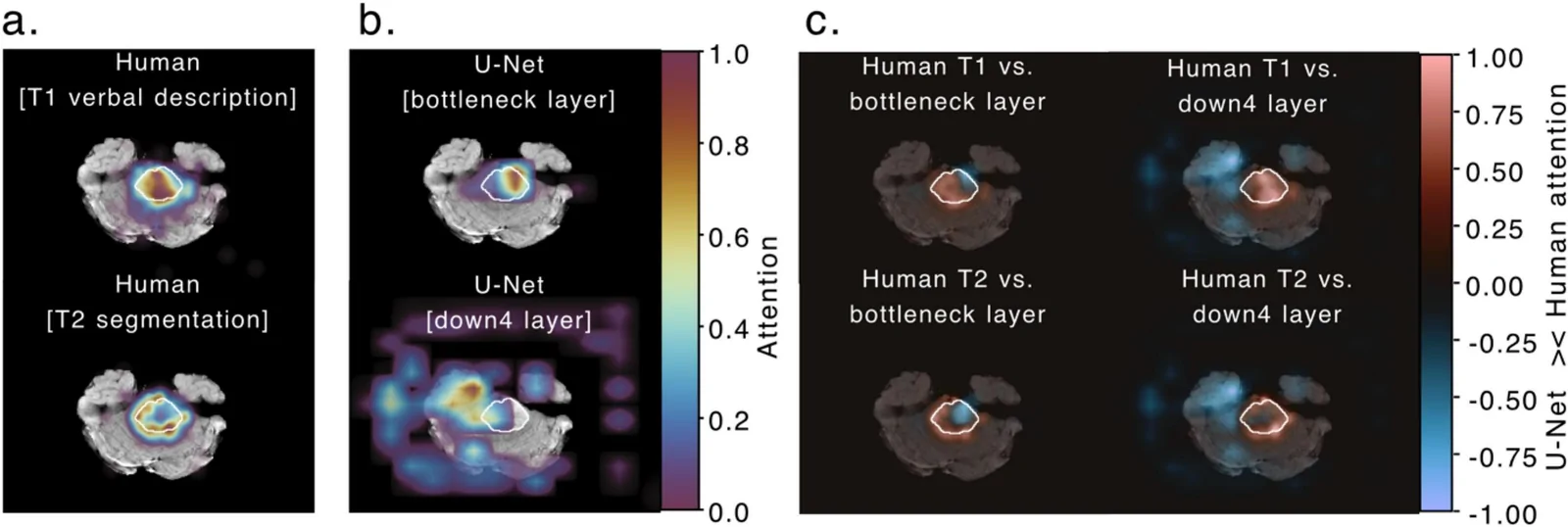
Comparison of human attention and U-Net saliency maps for Task 1 and Task 2 against two representative U-Net layers for a sample scan. The bottleneck layer saliency is displayed due to its high overall similarity to human attention, while the down4 layer saliency is shown for its low similarity to human attention. Overlaid in white is the ground truth tumour outline. (a) Human attention: Average human attention maps aggregated across participants. The top panel shows Task 1; the bottom panel shows Task 2. (b) Computer saliency: Saliency maps extracted from the deep learning model. The top panel shows the bottleneck layer; the bottom panel shows the fourth down layer. (c) Difference between human attention and computer saliency (human - computer) for the bottleneck (left) and down4 (right) layers. Positive values (red) indicate regions with higher human attention; negative values (blue) indicate regions with higher model saliency. The top panel shows Task 1; the bottom panel shows Task 2. (For interpretation of the references to colour in this figure legend, the reader is referred to the web version of this article.)

When compared, human gaze attention overlapped most with U-Net bottleneck saliency (average Dice = 0.6), followed by the final two upsampling layers and summed layers (Fig. 4). The downsampling layers, particularly down4, showed the lowest overlap (Dice = 0.1–0.2; Table S9).

Qualitative assessment of attention maps revealed that human observers typically explore the entire tumour during Task 1, similar to U-Net bottleneck layers. During Task 2, human attention tracked the tumour boundaries, while the fourth downsampling layer saliency maps often highlighted extracranial regions, explaining their poor overlap. Correlation analysis revealed that only the higher attention/saliency overlap between human Task 2 attention and down3/down4 saliency was moderately related to better human segmentation performance (|r| > 0.5, *p* < 0.05).

## 4 Discussion

In this study, behavioural and image-derived features showed potential for predicting inter-observer variability in manual tumour delineation, a widely accepted proxy for manual segmentation uncertainty and a key contributor to geometric deviations in contour-based radiotherapy planning. By quantifying and localizing this uncertainty, our approach provides a spatially resolved map for contour quality assurance and robust margin designs.

For the prediction of segmentation uncertainty, behavioural signals provided complementary information to image-derived features. Here, shorter saccade amplitudes and higher saccade velocities were associated with higher uncertainty, consistent with previous work [38] that linked saccadic patterns to uncertainty in object detection. In contrast, whereas Quetard et al. [39] reported slower cursor velocity to be associated with greater within-subject uncertainty in object detection, our results showed an association between faster cursor movements and increased uncertainty. This may reflect differences in task design and uncertainty computation. In our setting, faster mouse movements may indicate a speed-accuracy trade-off, reducing precision and increasing inter-observer variability.

Our predictive analyses support the potential generalisability of identified signals across participants and scans. The random forest explained 39% of variance in leave-one-out cross-validation, performance comparable to the mixed-effects fixed effects alone (marginal R^2^ = 0.30), yet without access to participant- or scan-specific intercepts, indicating that the features carry generalizable predictive information. This suggests that individual segmentation strategies, reflected in behavioural metrics, could support the uncertainty identification when combined with image information.

The moderate predictive performance reflects a structural feature of our framework: behavioural inputs capture intra-observer process indicators, whereas the response variable explicitly captures inter-observer variability, asymmetry which has previously been attributed to a shared origin in image-level ambiguity [3]. Complete disentanglement would require self-reported confidence and repeated delineations by the same observer.

Notably, the regression model yielded the most accurate uncertainty predictions in regions where human attention and U-Net saliency diverged. U-Net layers operate at different spatial scales, shallower layers encode fine-grained detail, whereas deeper layers encode coarser contextual information, and while not directly comparable, divergence from human attention may provide complementary information for contour quality assurance.

Our findings also reinforce known effects of task demands and expertise on gaze behaviour. Verbal descriptive tasks elicited broad, exploratory patterns, while the delineation tasks elicited a more focused gaze directed along tumour boundaries. High-performing participants showed expert-like gaze patterns, with fewer but longer fixations, higher saccade velocities, and a lower fixation entropy, consistent with prior work on expert visual search in radiology [24], [40], [41].

The proposed framework represents a step toward uncertainty-aware contour assessment in radiotherapy. While probabilistic clinical tumour volume (CTV) definitions are emerging; they focus on the modeling of biological infiltration [13], [14], while ignoring observer uncertainty. Previous work has shown that diffuse glioma borders are inherently ambiguous on standard MRI [42]. This suggests that regions of unclear definition may spatially coincide with the infiltration regions, making uncertainty annotation a meaningful signal for guiding a non-uniform CTV expansion. In practice, eye- and mouse-tracking could run passively during routine segmentation, returning the contour with a co-registered uncertainty map to be considered for downstream dose distributions. Additionally, such features can be derived independently of anatomical location, tumour type, or imaging modality, demonstrating the potential for this framework to scale to diverse delineation tasks.

While our results suggest potential for automated uncertainty prediction, several limitations should be acknowledged. As a pilot study designed to assess the feasibility of quantifying uncertainty from behavioural and image-derived signals, we prioritized task duration over clinical realism: participants segmented single axial T2-FLAIR slices, without zooming, scrolling, or switching between contrasts and at a sub-standard resolution (<512 × 512). Future research should reflect clinical conditions more closely, incorporating standard segmentation software and screens, multiple contrasts and realistic time pressure, and should focus on domain experts rather than self-reported medical professionals with varying backgrounds.

On the feature level, the behavioural set could be extended to include pupil dilation [43], blink rate [38], and gaze-cursor coordination [44], alongside tool-interaction measures (e.g., brush size, editing time, contrast switching, or scrolling behaviour [45], [46]). Verbal narration could further reveal reasoning and confidence in real time. On the imaging side, incorporating radiomic descriptors of local texture and heterogeneity or U-Net-derived uncertainty maps could strengthen the image-derived component. The uncertainty metric itself could be refined through self-reported confidence, or by adopting alternative distance metrics, such as the bidirectional local distance, to better capture directional asymmetries in contour disagreement. Finally, this framework could extend to AI-assisted editing workflows, where behavioural signals would reflect edit-specific interactions.

This study supports viewing expert segmentations as continuous rather than absolute ground truth. By enriching manual contours with quantified uncertainty, our work provides a foundation for better capturing diagnostic ambiguity, enhancing trust, and facilitating more case-adaptive, robust radiotherapy planning following future clinical dose-volume validation.

## Declaration of generative AI and AI-assisted technologies in the manuscript preparation process

During the preparation of this work the author(s) used ChatGPT (GPT4o mini, GPT5.2) in order to improve flow and conciseness. After using this tool/service, the author(s) reviewed and edited the content as needed and take(s) full responsibility for the content of the publication.

## Data sharing

Imaging data is publicly available at https://synapse.org/Synapse:syn51156910 upon request. The acquired eye and mouse tracking data is available upon request from the senior authors (contact Prof. Sarah Brüningk, University of Bern, sarah.brueningk@unibe.ch; Prof. Arzu Coltekin, University of Applied Sciences and Arts Northwestern Switzerland, arzu.coltekin@fhnw.ch).

## CRediT authorship contribution statement

**Nina Baumgartner:** Writing – review & editing, Writing – original draft, Visualization, Validation, Methodology, Formal analysis, Data curation. **Daria Laslo:** Writing – review & editing, Writing – original draft, Visualization, Validation, Supervision, Methodology, Investigation, Formal analysis, Conceptualization. **Laura Fontanesi:** Writing – review & editing, Methodology, Formal analysis, Data curation. **Catherine R. Jutzeler:** Supervision, Resources. **Arzu Çöltekin:** Supervision, Software, Resources, Project administration, Funding acquisition. **Sarah Brüningk:** Supervision, Resources, Project administration, Funding acquisition.

## Funding

DL was supported by the Chad Tough Defeat DIPG Foundation under 1477715. SB was supported by the Swiss National Science Foundation under TMSGI3_225913. AC and LF were supported by Google Faculty Research Award (2014_R2_742).

## Declaration of competing interest

The authors declare that they have no known competing financial interests or personal relationships that could have appeared to influence the work reported in this paper.

## Appendix A Supplementary data

Supplementary material Supplemental clinician survey, methodological details on the segmentation model and experimental protocol, and additional analyses of participant, behavioural, and point-level annotation variables (Figs. S1–S12, Tables S1–S9).
