# Supplementary material for: Toward uncertainty-aware manual delineation of brain tumours using eye-tracking and image-derived features
Source: Phys Imaging Radiat Oncol. 2026 Aug 4;40:101056. doi: 10.1016/j.phro.2026.101056 (PMC13495590; doi:10.1016/j.phro.2026.101056)
Supplement: Supplementary file 1 [file mmc1.pdf]

Supplementary Material

A. Supplemental Clinician Survey

Methodology

To complement the experimental study, a brief survey was conducted to assess how medical professionals perceive the value of incorporating uncertainty annotations into manual tumor segmentations. This survey targeted a different group of participants than those involved in the main experiment. It was distributed through radiology networks and sent directly to leaders of radiology departments at various Swiss hospitals. The survey collected demographic and professional background information, alongside participants' opinions on the potential benefits of uncertainty annotations. An optional section invited written feedback and offered the opportunity to provide contact details for follow-up after the study's conclusion. Participants gave informed consent by submitting the survey, agreeing to the use and publication of their responses in this research.

Results

A total of 18 clinicians completed the survey whose results are presented in Fig. S1. Their backgrounds (in decreasing order of mentions) included radiology, radiation oncology, neuroradiology, interventional radiology, radiotherapy, musculoskeletal radiology, and prostate and female pelvic MRI. 12 participants (66.7 %) reported more than 11 years of experience, followed by three participants (16.7 %) with 6-10 years of experience. Three participants further stated to have no experience in radiology, as their official roles are radiotherapist and radiation oncologist. The average usefulness rating of uncertainty-annotated manual segmentations was 7.33. The most stated were 9 and 7. The range was 2-10.

Regarding potential applications, nine clinicians expressed a desire to use uncertainty information in radio-therapy planning, followed by eight who valued it for communication with colleagues. Other uses mentioned, in descending order, included AI tool development, resident or student training, surgery planning, communication with patients, and diagnosis. Three clinicians stated they would not use such information.

Concerning trustworthiness, 50% of clinicians indicated they would trust a colleague's segmentation more if it included an uncertainty measure, while 38.9% were unsure, and 11.1% would not increase their trust. Similarly, 61.1% said they would trust AI-generated tumor segmentations more with added uncertainty information, 33.3% were uncertain, and 5.6% would not trust them more. This highlights the greater trust placed in human annotations compared to those generated by AI. However, in both cases, including uncertainty annotations would enhance overall trustworthiness.

To explore the perceived utility, implementation, and concerns regarding uncertainty in tumor segmentation, a set of optional open-ended questions was included in the survey. Responses are thematically analyzed and grouped for clarity.

- **Communication of Uncertainty:** Participants (n = 11) were asked how they believe uncertainty in tumor segmentation should be communicated (e.g., confidence intervals, color-coded maps, probabilistic contours). The most frequently suggested formats were probabilistic contours and color-coded uncertainty maps, with several respondents highlighting the value of these formats for tasks such as radiotherapy planning or longitudinal follow- up. Other suggestions included confidence intervals and expanding PTV (planning target volume) margins. One respondent emphasized the importance of visual overlays directly on medical images, while another was unsure of how to best communicate uncertainty.

- **Use in Research:** When asked how they would use uncertainty-annotated segmentations in research (n = 7), several participants noted potential applications in model development and evaluation. These included using un- certainty maps to retrain models, assess prognostic value in follow-up studies, and identify limitations or biases. One respondent mentioned correlating annotations with surgical explants. Others were unsure or would use the annotations "as is."
- **Use in Clinical Practice:** In the clinical context (n = 10), uncertainty annotations were viewed as potentially valuable for adjusting PTV margins, guiding follow-up intervals, and identifying the need for intervention. Some respondents indicated the utility of uncertainty for optimizing tumor contours, highlighting ambiguous areas, and reducing clinician review time. One noted its role in facilitating interdisciplinary communication with surgeons and radiotherapists. However, a few respondents expressed indecision or stated they would use the annotations without modification.
- **Concerns About Implementation:** Concerns regarding the implementation of uncertainty annotations (n = 11) varied. Several participants expressed no concerns, while others emphasized the need for clear presentation, clinical relevance, and ease of integration into existing workflows. One respondent raised concerns about false positives in cases with otherwise high-quality segmentations, urging the need for supervised review to reduce misleading doubt scores. Another stressed that the tool should be fast and intuitive to encourage adoption.
- **Additional Comments:** Five respondents offered further suggestions. These included a proposal to integrate uncertainty into tumor volume doubling time calculations and an offer to share published results on the topic. Others had no additional comments.

B. Supplementary Information on the Ground Truth Segmentation Masks

In this experiment, we make use of the ground truth segmentations provided as part of the BraTS23 pediatric segmentation challenge [26]. These were generated starting from automated segmentations that were manually reviewed by American Society of Neuroradiology volunteer neuroradiology experts of varying rank and experience, following the established guidelines as detailed in Fathi Kazerooni et al. [26]. These were further reviewed by three attending board-certified neuroradiologists. They were either accepted or sent back for refinements until a subsequent acceptance.

C. Scan Selection and U-Net Implementation

For tumor segmentation, a standard U-Net architecture is developed internally, relying on the original implementation [23], using the T2-FLAIR modality as single-channel input. The network is composed of four encoder blocks and four decoder blocks, connected by a bridge. Each encoder block contains two 3x3 convolutions, followed by a ReLu (rectified linear unit) activation function and a 2x2 max-pooling layers. The final layer reduces the dimensions of the features by half. Each decoder block contains a 2x2 transpose convolution which doubles the spatial dimensions followed by two 3x3 convolutions and ReLu. The final decoder block contains a 1x1 convolution with a sigmoid activation function. The result of the final layer is a classification of each pixel either as a tumor or a healthy pixel.

Given limited pediatric data, the model is trained using a transfer learning approach, using the BraTS23-GLI (B. H. Menze et al., "The Multimodal Brain Tumor Image Segmentation Benchmark (BRATS)," in IEEE Transactions on Medical Imaging, 2015) and BraTS-PED [26] datasets. The model is initially trained on the adult training subset (n=1251), then the weights of the first two encoder block are frozen and the network is trained for a further 3,000 epochs on the training BraTS23-PED subset (n=129). We report an average Dice of 0.83 for the hold-out test set of BraTS23-PED (495 slices from 6 patients) with a standard deviation of 0.30. We note that the samples used in this study are a subset of the hold-out test set and have not been seen during the training of this model.

The “difficult” examples are selected as to be below the 25th percentile (Dice=0.81) based on the Dice score distribution over all BraTS23 test samples.

Uncertainty quantification was done using test-time augmentation, with flips and 90 and 180 degree rotations.

D. Supplementary Information on the Experimental Setup and Procedure

All experiments were conducted on site during the European Congress of Radiology 2025 at the Austria Center Vienna. The study was part of “The Perception Lab – Medical Image Perception at ECR” (https://connect.myesr.org/course/the-perception-lab-medical-image-perception-at-ecr-2/).

Experiments were carried out in a dedicated, enclosed space to minimise external distractions (see Supplementary Fig. S9). Participants were recruited in the hallway after ensuring a completed medical education and confidence with the anatomy of the brain and tumour segmentations. Participants were then taken to the back of the area, where they could enter the experiment room. Participants were seated in front of a display, with their head stabilized using a headrest. They interacted with the experimental interface using a computer mouse. Video and audio recordings were obtained concurrently (with written consent). Sequentially in order of appearance, the participants were assigned to one of the three experimental groups to ensure that there is an equal number of raters per scan and to randomise the group assignment. The goal was to achieve 30 completed experiments (10 per experimental group, 12 scans with 10 raters each) by the end of the conference.

The experimenter remained positioned at the experimenter’s computer, generally outside the participant’s field of view, and monitored the experiment via two screens: one displaying the eye-tracking software and gaze data, and another mirroring the participant’s visual display. Verbal instructions were provided only when task statements presented on the screen required clarification. In most sessions, only the participant and the experimenter were present in the room. In a small number of cases (n = 2), participants requested the presence of an additional person; such individuals were instructed not to interfere with the experimental procedures.

To standardise the experimental procedure, a data collection protocol was compiled and followed meticulously in every experiment.

Upon arrival:

1) **Sign the informed consent sheet:** The “Participant Information Statement and Consent Form” contained information about the purpose of the study, a description of the study and associated risks, confidentiality and disclosure information, information about compensation, and the consent statement. The participants were asked to explicitly state that they consent to participating in the study.
2) Hand over the forehead sticker for eye-tracking and let the participant place it in the correct location.
3) Together fill in the participant information and provide information if needed (i.e. years of experience = after completion of medical education, at start of residency).
4) Adjust the height of the head mount and focus of the eye-tracker.

Before starting the experiment:

5) Check the eye-tracking time resolution and calibration settings on the host laptop.
6) Start calibration and validation (press “Enter” when the participant fixates the calibration point).

7) Explain the two tasks and that they will be repeated in the same order for four stimuli. Highlight that no clicking of the mouse is required. Highlight the task duration and that they are free to move on to the next task whenever they are done.

During the experiment:

8) Give only very brief instructions throughout the tasks (i.e. "now describe" (Task 1), "now outline" (Task 2)).

After the experiment:

9) Thank the participant and let them choose a small gift (chocolate or pen).
10) Ask the participant whether they wish for a "debriefing" and if they do, show them the scans again, this time including the ground truth segmentation masks.
11) Ask the participant whether they wish to be informed about the publication of this work.
12) Take notes about "Speech quality" (volume, understandability), "Language quality" (level of English, some superficial content assessment), "Eye quality" (notes about how the eye-tracking software was doing), and "Mouse quality" (notes about segmentation) as well as any other remarks.
13) Disinfect the whole setup.

During the experiment, the participants saw the following instructions on their own screens:

1) **Calibration of the eye-tracker:** a random sequence of five points on the screen, which need to be fixated on until calibration is performed.
2) **Introduction:** the following text was displayed on the screen:

*"The total time for this experiment is 20 minutes. You will assess 4 single axial slice **T2FLAIR brain MRIs** of paediatric diffuse midline glioma patients. Note that only a single axial slice and a single contrast is provided. Zooming or windowing is not enabled.*

For each image, you will complete two tasks:

*1. **Description** – Obtain an overview of the image first. Then describe (verbally) the tumour's spread, boundaries, and any other notable features of relevance if tasked with the segmentation of the whole tumour area in the image.*

*2. **Delineation** – Use the mouse cursor to outline the tumour's borders, as if demonstrating them to a trainee.*

You will have up to 1 minute for each task.

Press Enter to begin."

3) **Task 1 (description):** the following text was displayed on the screen:

"Have a look at the image first. Then describe your thoughts in words, focusing on the tumour boundaries, extent, and any other relevant features that you would consider relevant for a tumour segmentation task.

***Do not use hand gestures or the mouse cursor** to point at specific areas.*

*You have **up to 1 minute** for this task.*

*Press **Enter** to begin, and press **Enter** if you finish before the time is up."*

4) **Task 2 (delineation):** the following text was displayed on the screen:

*"Use the mouse cursor to **slowly** outline the tumour, as if demonstrating its borders to a trainee. If needed, you may outline the borders multiple times if you would like to change your previous opinion.*

Focus on precision and clarity in your delineation.

You have ***up to 1 minute*** *for this task.*

Press ***Enter*** *to begin, and press* ***Enter*** *if you finish before the time is up."*

5) Repeat 3+4 three more times (go through all four scans).
6) **Outro:** the following text was displayed on the screen:

*"**Thank you for your participation.** We appreciate your time and expertise, and we wish you a great continuation of the conference!*

If you have any questions or feedback, please let us know."

E. Hyperparameter Tuning of Random Forest

The number of estimators (50, 100, 300, 500), the maximum tree depth ('None', 10, 20, 50), and the maximum number of features ('sqrt', 'log2', 'None') were optimized for minimal root mean squared error (RMSE) (top value underlined).

F. Classifier for Discrete Uncertainty Predictions

Methodology

In addition to the regression model, a classifier was trained to predict the binarized variability (top vs. bottom 40%, excluding the middle 20%) to mitigate mean-focused predictions. The resulting model was evaluated at the testing group, participant, and stimulus levels. Performance was assessed using accuracy, precision, recall, F1, and AUC metrics, computed per outer fold and aggregated to assess consistency.

Results

The classifier trained on the combination of behavioral and image-based features achieved an accuracy of 74.8% across all testing folds (precision = 0.7, recall = 0.9, F1 = 0.8, ROC-AUC = 0.9) for predicting high vs. low variability. When applying the model trained on the top/bottom 40% of variability values to the full testing set, including the intermediate 20% of samples, the model's predicted probabilities for the high-uncertainty class correlated strongly with true uncertainty (Spearman $r = 0.6$, $p < 0.001$).

Furthermore, the feature importance analyses supported the regression findings. The top predictors were again image-based, with higher image entropy and greater saliency in encoder/downsampling layers associated with increased uncertainty. Interestingly, saliency in the upsampling layers, which previously predicted higher uncertainty in the regression model, was here linked to lower uncertainty. SHAPley values also highlighted behavioral predictors of high uncertainty, including greater fixation density in Task 1, faster mouse velocity, and faster saccade speeds, aligning with trends observed in the regression model.

Classifier performance varied across the 28 participants (accuracy range: 0.4–1.0) and 12 stimuli (range: 0.3–1.0). However, variation in participant-level model performance was not associated with age, expertise, experience, or any other measured participant-related variable (Spearman correlation with performance metrics, $p > 0.05$). At the stimulus level, better classifier precision and F1 scores were linked to lower overlap between Task 2 human attention maps and computer saliency in the third and fourth Grad-CAM

upsampling layers (Spearman $|r| > 0.5$, $p < 0.05$). This suggests that greater distinction between human and model saliency may support more accurate uncertainty classification at the stimulus level.

G. Supplementary Information on the Participants

Regarding the participant metadata, only specialty was a predictor of performance. Grouping participants into four clusters (oncology, general radiology, neuroradiology, non-radiologist/non-oncologist) reveals a clear gradient. Neuroradiology/brain MRI specialists achieve the highest group mean (~0.76). The two top performers overall are both neuroradiology/head specialists. The non-MRI/no-DMG-knowledge group is by far the weakest (mean ~0.45), driven mainly by two participants (mean 0.18, a CT/3D specialist who explicitly had no idea about DMG and mean 0.24, a radiographer rather than radiologist). Nonetheless, one participant in that group performed much better (mean 0.74), likely because they are a physician with general diagnostic exposure. General radiology and oncology sit in the middle (~0.72), performing similarly to each other.

The apparent continent effect (Europe 0.72 > Asia 0.70 > Americas 0.55) is almost entirely an artefact of one participant (USA, mean 0.18) dragging the Americas group down. If this participant is removed, the Americas mean rises to ~0.72, making all three continents essentially equivalent. There is no meaningful continent effect independent of specialty mismatch. Similarly, country-level variation does not reflect genuine geographic patterns but it reflects the individuals assigned to each country, most of which have n=1. Countries appearing high (Switzerland mean 0.82, Romania 0.82, Croatia 0.82) simply happen to contain participants who were competent and well-matched to the task. Countries appearing low (USA 0.18, Belarus 0.59, Finland 0.57) contain individuals with confounding issues such as domain mismatch, partial blindness, or instability (some fully missed outlines). No country-of-training effect can be inferred from this sample size.

Furthermore, there is no clear relationship between years of experience (after finishing medical education) and segmentation performance. There are highly experienced participants with poor Dice (24y + Dice 0.18; 30y + Dice 0.61; 30y + Dice 0.65) and relatively junior participants with strong Dice (2y+ Dice 0.83; 2y + Dice 0.82; 5y + Dice 0.85). The key confounder is that experience in the wrong specialty is not useful. Within neuroradiology specifically, there is a weak positive trend, but the sample is too small to draw firm conclusions.

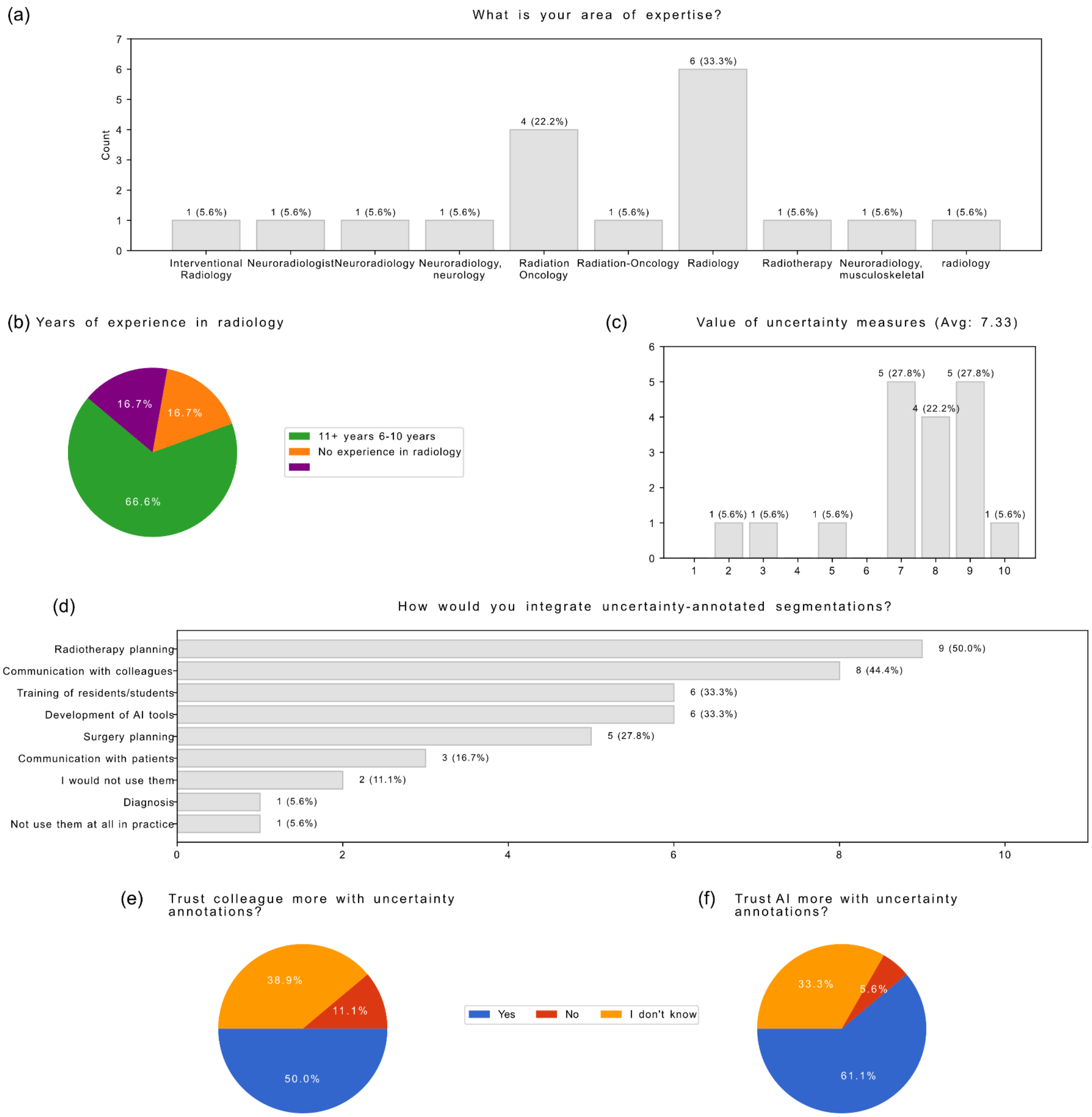

Fig. S1. Survey Analysis of Clinician Expertise and Perceived Utility of Uncertainty Measures. Data reflects responses from medical specialists (n = 18). (A) Primary areas of expertise are dominated by Radiology and Radiation Oncology. (B) Clinical experience in radiology; 66.7% of the cohort possesses over 11 years of experience. (C) Perceived value of including uncertainty measures in manual tumour segmentations, scored on a Likert scale (1–10). The mean utility score was 7.33. (D) Potential workflow integrations for uncertainty-annotated data, with Radiotherapy planning and intra-departmental communication being the most cited use cases. (E) Impact of uncertainty measures on trust in human-generated segmentations, where 50% of respondents indicated increased trust. (F) Impact of uncertainty measures on trust in AI-generated segmentations; 61.1% of respondents reported higher trust when uncertainty data is present.

(a) (b)

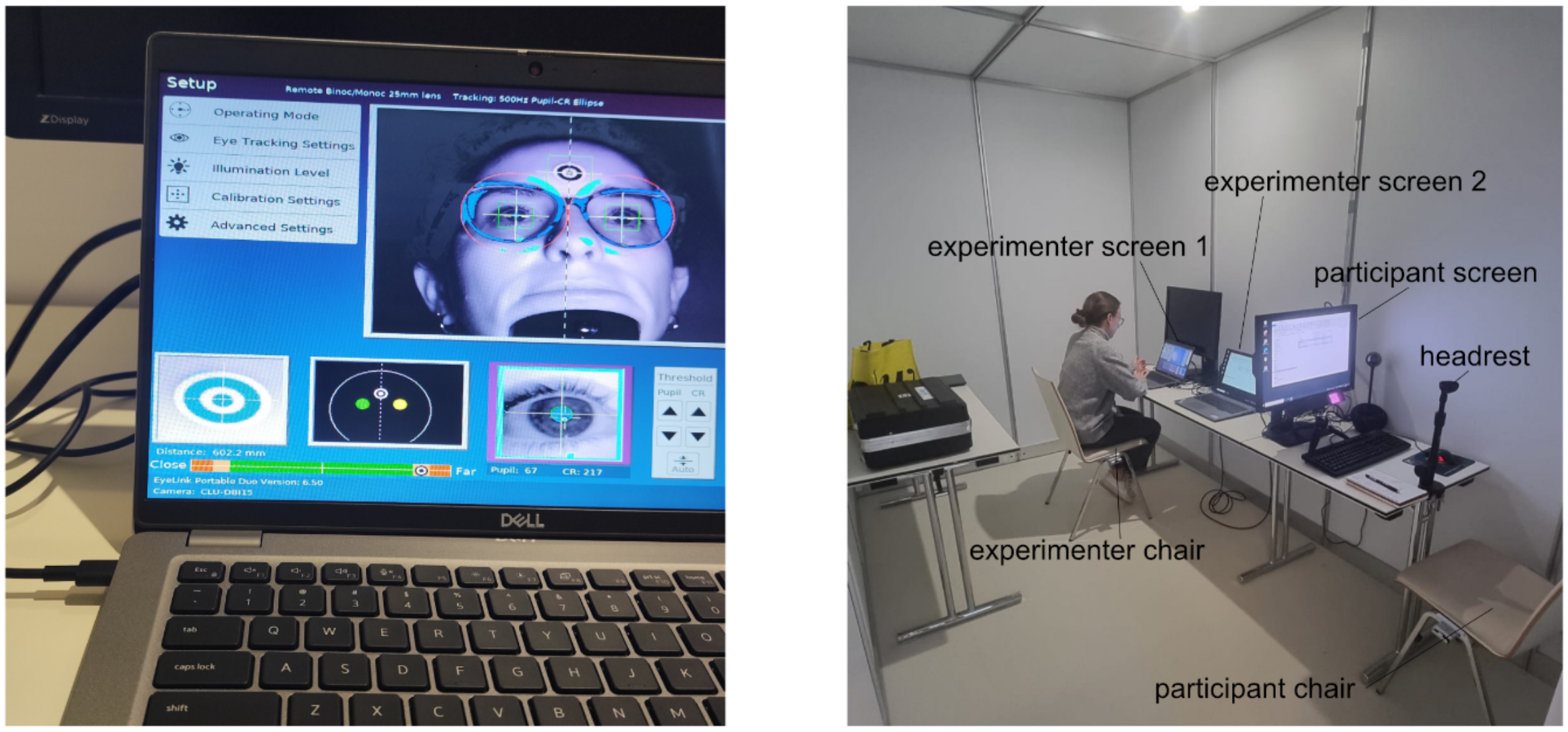

Fig. S2. Experimental setup and eye-tracking configuration. (a) Example of the eye-tracking calibration interface, showing real-time detection of facial features, pupil position, and gaze tracking parameters. (b) Photograph of the experimental environment within the enclosed testing space. The participant was seated in front of the stimulus display with head stabilization provided by a headrest. The experimenter was positioned behind the participant and outside their field of view, monitoring the session via two screens: one displaying the eye-tracking software and gaze data, and the other mirroring the participant's display. The layout of participant and experimenter seating, as well as the placement of equipment, is indicated.

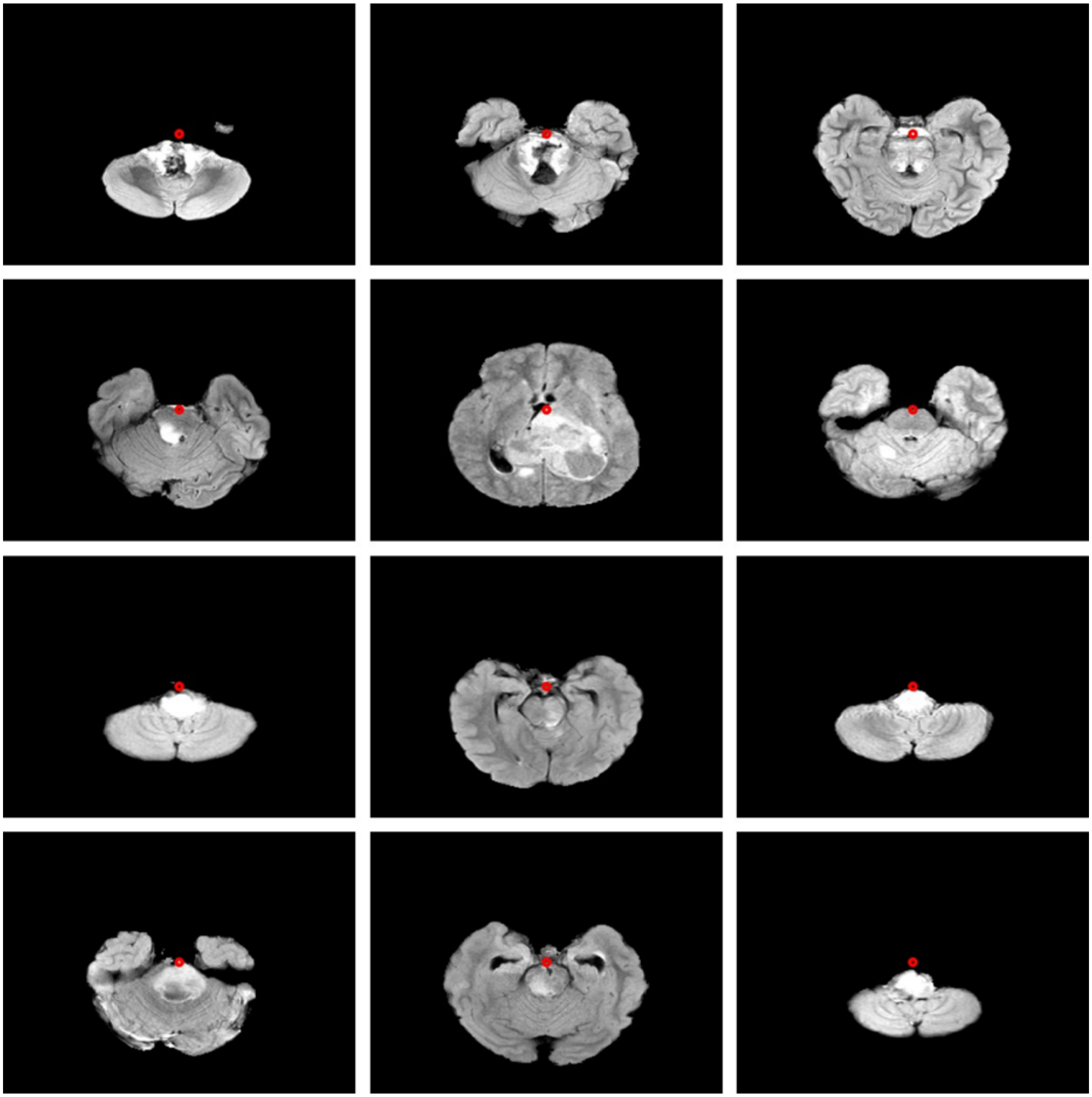

Fig. S3 **.** To annotate specific points of the mouse-track outline with eye-tracking data, a certain visual threshold in pixels around the point-of-consideration was defined. For that, both the human visual resolution, eye-tracker precision, and tracker calibration error were considered. At the given distance to the screen, the human visual perception corresponded to approximately one pixel. For the EyeLink Portable Duo, the typical accuracy corresponds to 0.25° to 0.5° of visual angle. For the calibration error, we accepted participants with a validation score of "GOOD" (worst point error < 1.5°, avg error < 1.0°). Furthermore, the spatial resolution is at 0.01° of visual angle. At a sampling rate of 2000 Hz, the noise levels (RMS) with a normal filter are expected to be at 0.03°. Given that the average error can thus be expected below 1° and the accuracy at 0.5° visual angle or lower, we can consider a window of radius 20 pixels (1° = 45 pixels), which is sufficiently small that the spatial resolution of the analysis is not compromised.

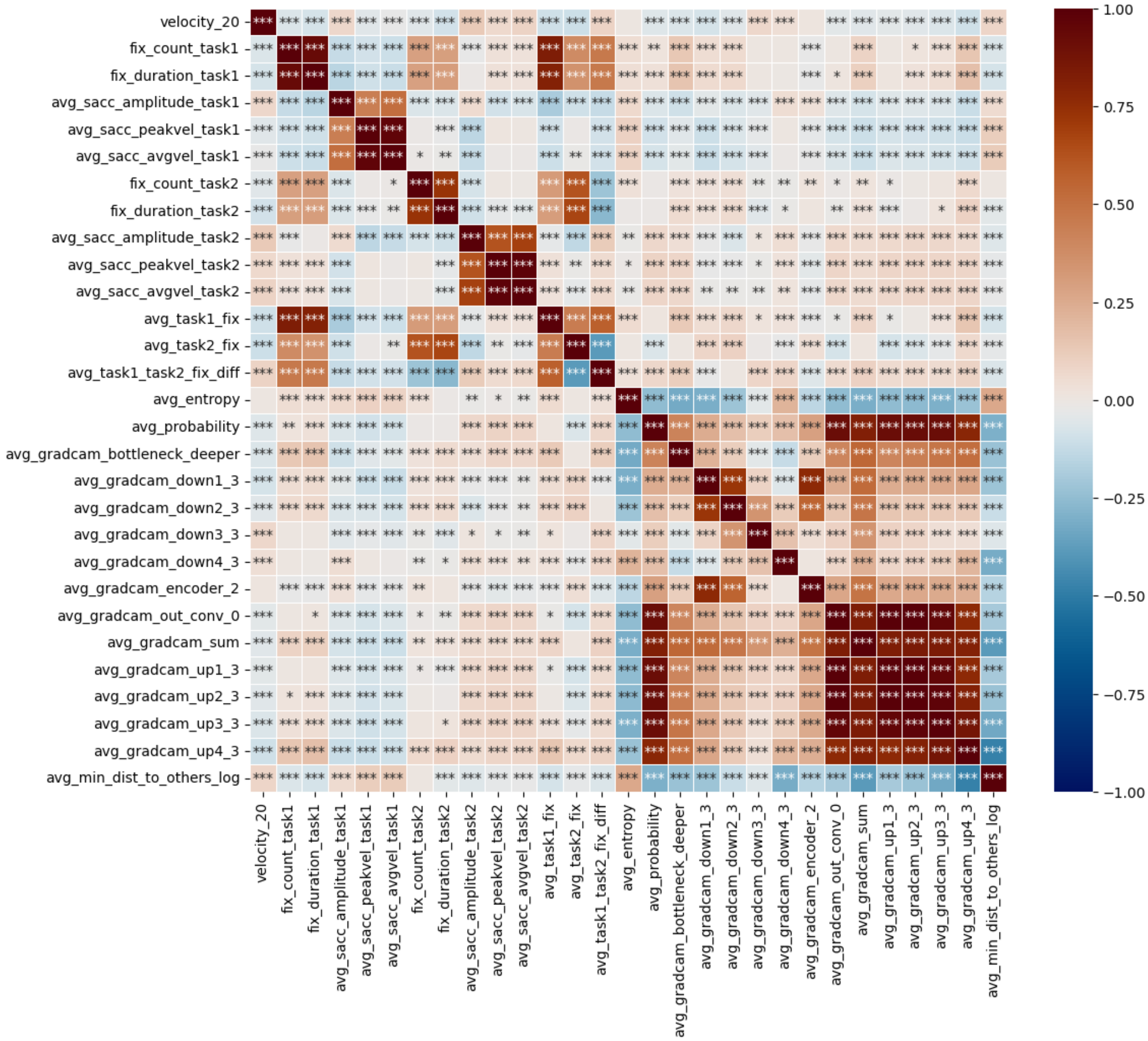

Fig. S4. Descriptive statistics of point-level annotation variables across all mouse-track outline points. Statistical significance is indicated by asterisks (*** $p < 0.001$, ** $p < 0.01$, * $p < 0.05$), with p-values corrected for multiple comparisons using the Benjamini–Hochberg False Discovery Rate (FDR) procedure.

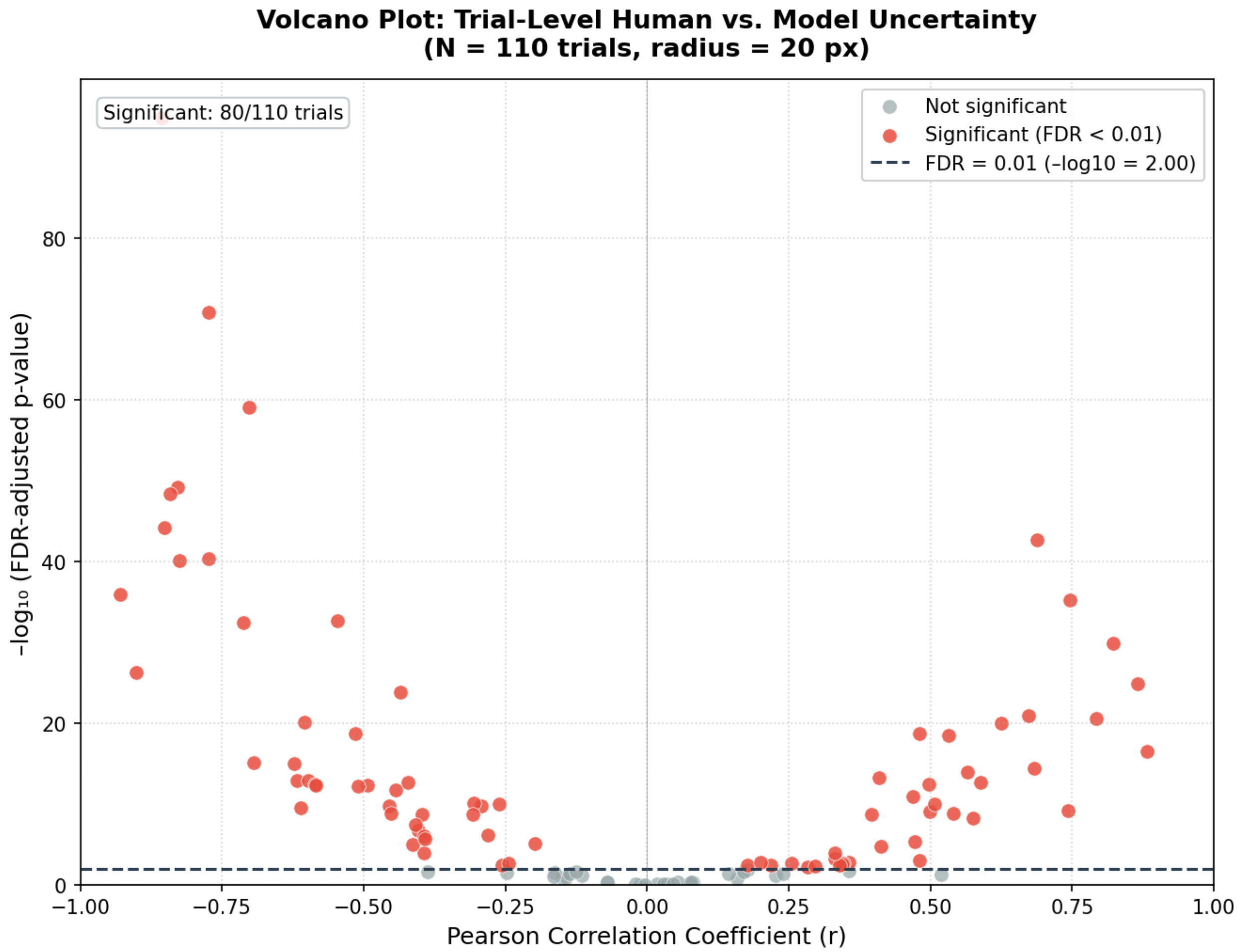

Fig. S5. Trial-level correlations between human and U-Net uncertainty. Volcano plot of the association between human perceptual uncertainty and U-Net-derived uncertainty (test-time augmentation) across N = 110 participant–scan trials, evaluated at annotation points within a 20 px radius. Each point is one trial, plotting its Pearson correlation (r) against significance ($-\log_{10}$ FDR-adjusted p-value); the dashed line marks FDR = 0.01. Significant trials (80/110) are shown in red, non-significant in grey. Although most trials reach significance, correlations split between positive and negative values with no consistent direction (median r = –0.016), indicating that human–model uncertainty associations are highly stimulus-dependent rather than systematic.

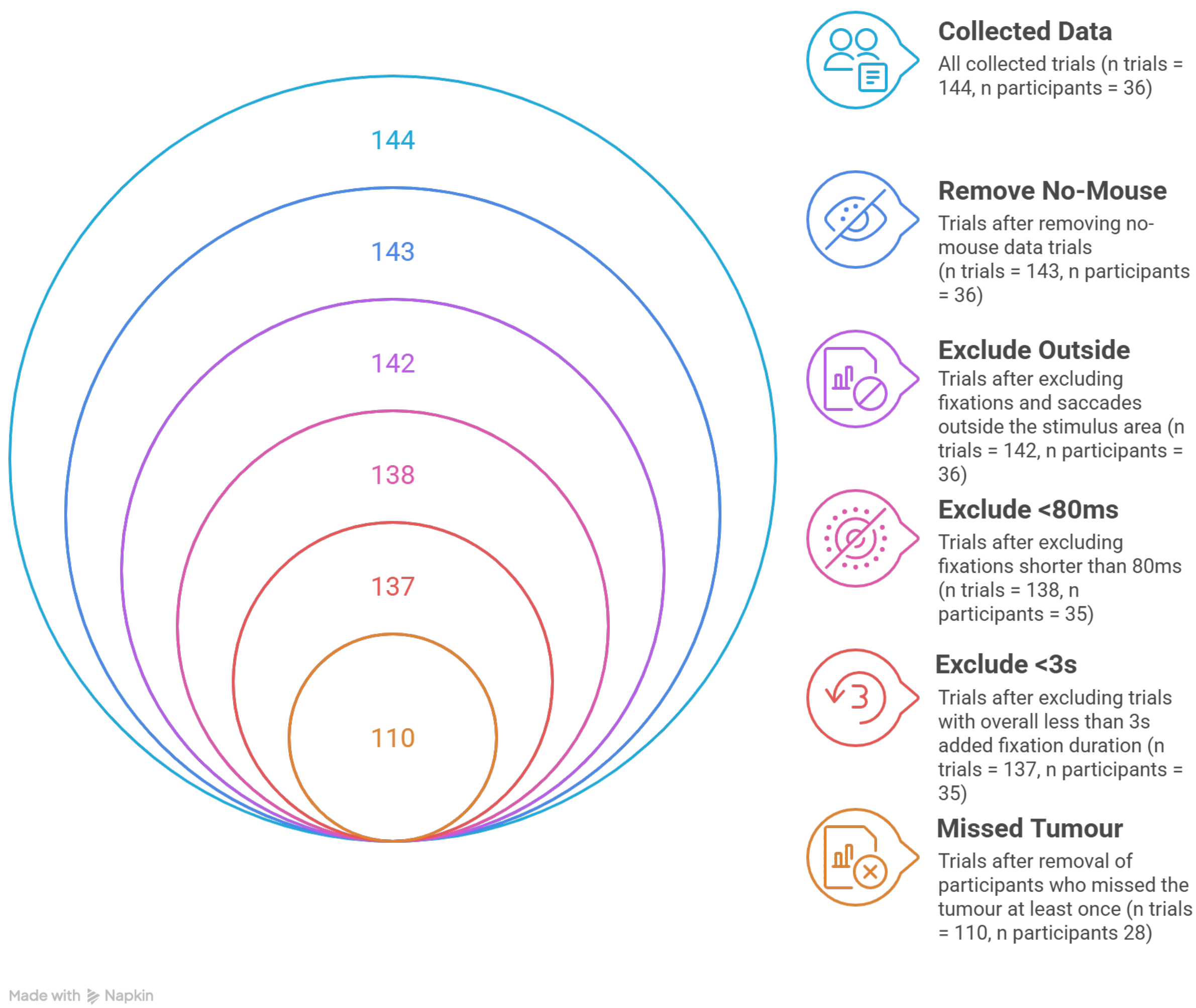

Fig. S6: Overview of the trial filtering and exclusion. To be noted that all of the excluded participants had no explicit primary expertise in neuroradiology and reported a below-average experience level (6.6 years compared to the 10.8 years overall average). Furthermore, three of these participants were noticeably nervous during the task.

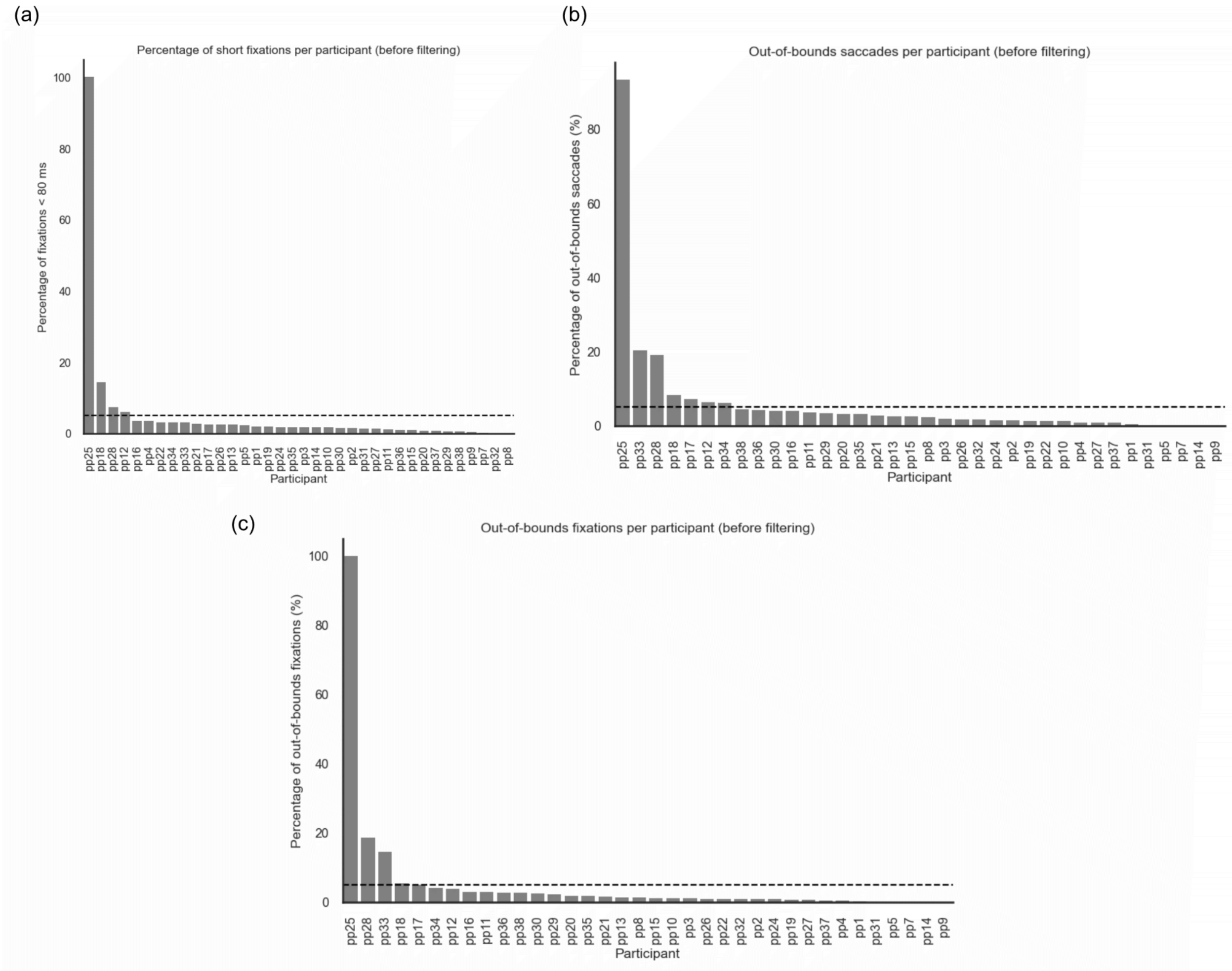

Fig. S7. An overview of (a) the percentage of short fixations (<80ms) per participant, highlighting the one participant who was excluded for having only short fixations, (b) out-of-bounds saccades, meaning saccades outside the area of the scan, and (c) out-of-bounds fixations, meaning fixations outside the scan, highlighting the same participant as before for also having all fixations outside the scan window. The dotted line highlights the 5% mark. pp25 was not noted to be distracted or nervous, hence the observed patterns are likely a technical artefact.

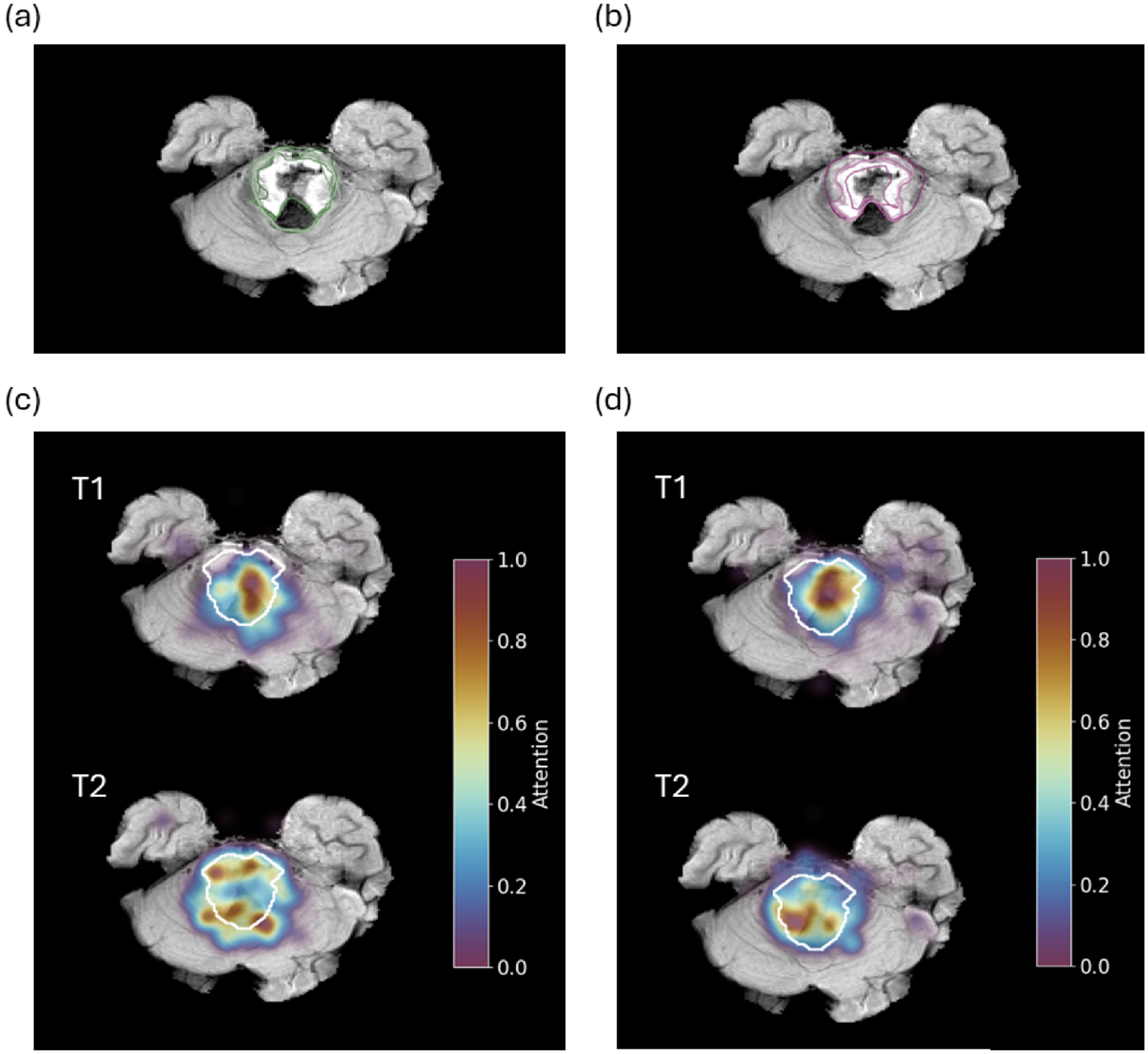

Fig. S8. Comparison of the visual attention between high-performing and low-performing participants. (a) All manual segmentations from high-performing participants. The different colors indicate outlines from different participants. (b) All manual segmentations from low-performing participants. The different colors indicate outlines from different participants. (c) Average human attention map from Task 1 (T1) and Task 2 (T2) across all high-performing participants. (d) Average human attention map from Task 1 (T1) and Task 2 (T2) across all low-performing participants.

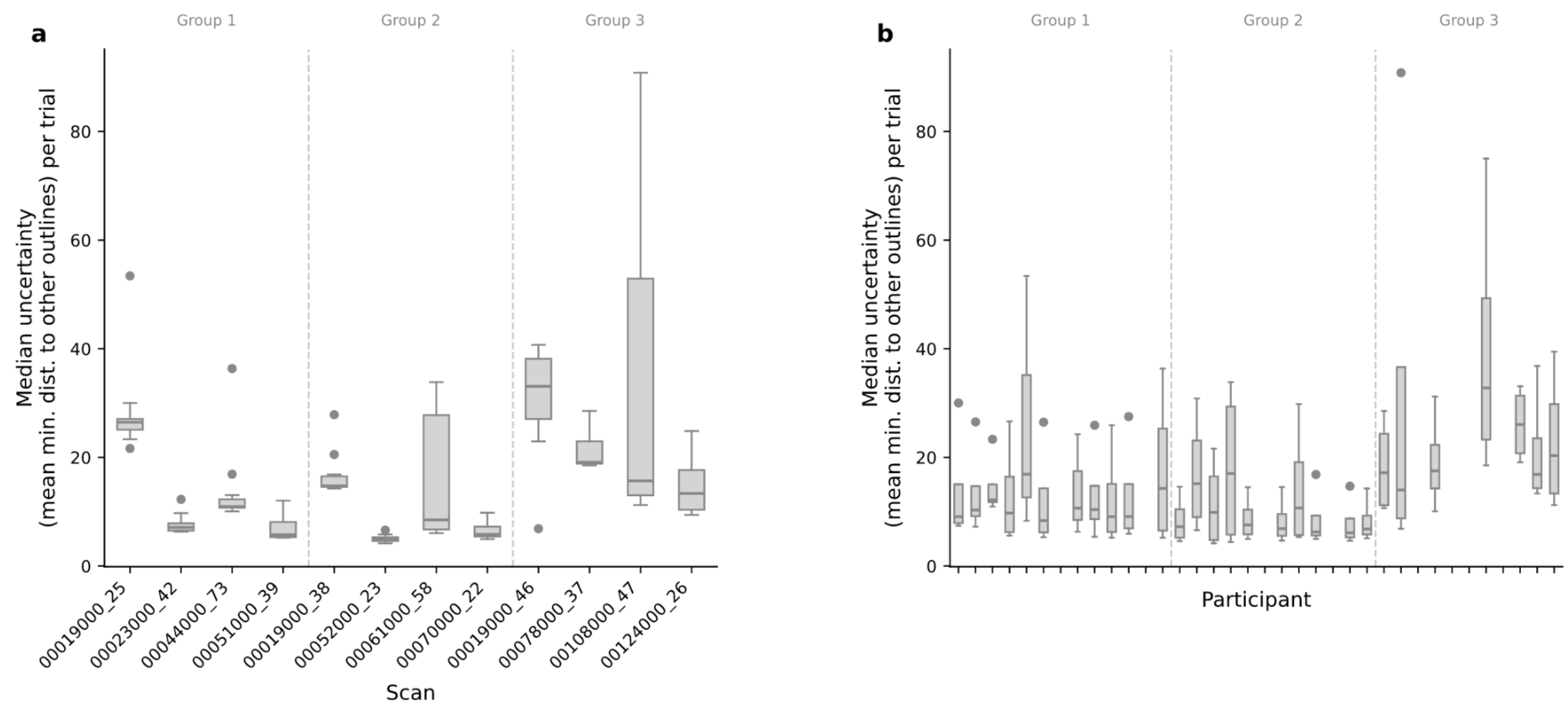

Fig. S9. The median uncertainty (mean minimal distance to all other outlines [pixels]) per trial (unique participant-scan computation) across (a) scans and (b) participants.

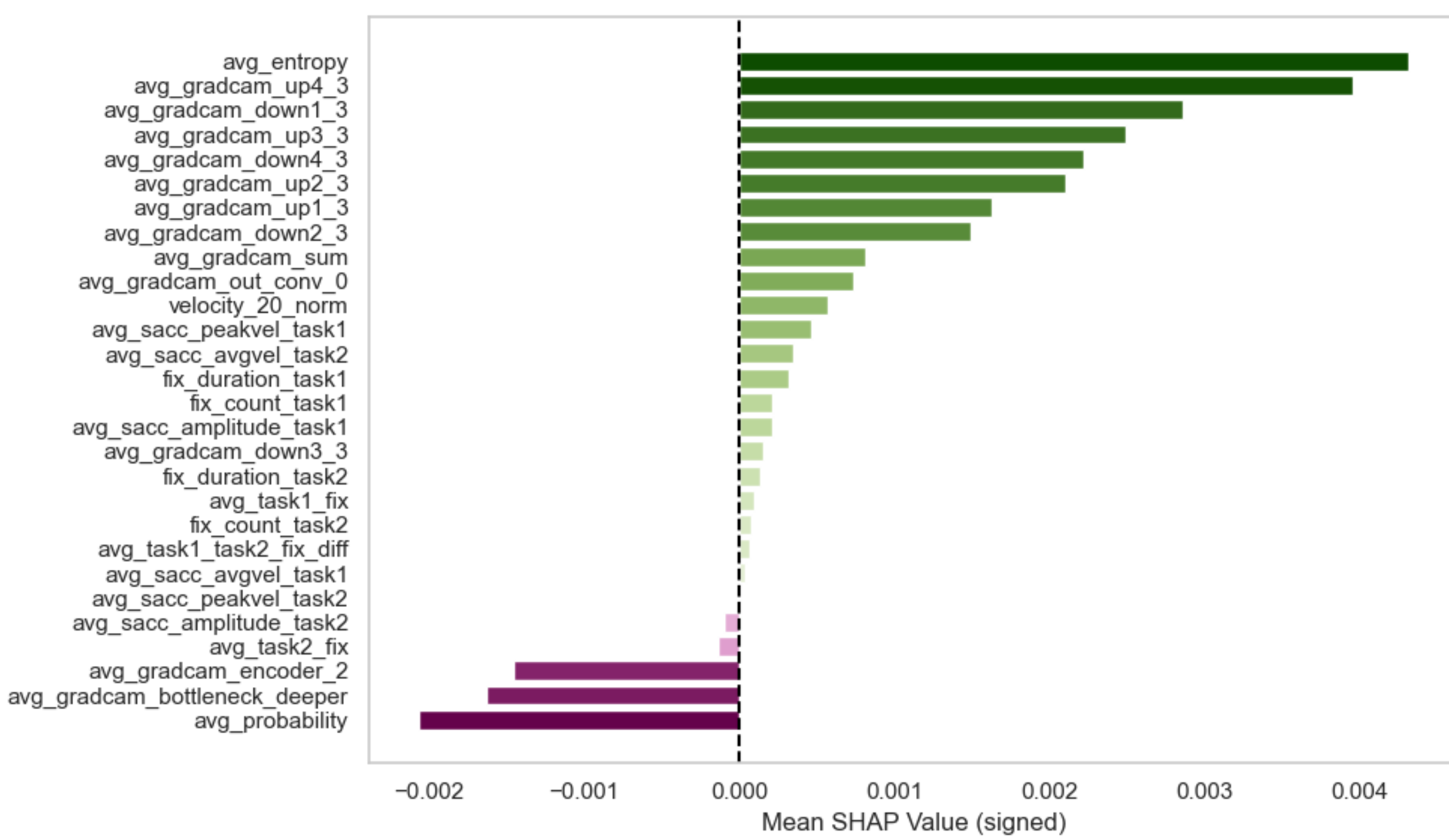

Fig. S10. Signed average SHAP (SHapley Additive exPlanations) values across the three outer folds for the regression model. Positive SHAP values indicate that higher variable values are associated with high

uncertainty. Negative SHAP values indicate that higher variable values are associated with lower uncertainty.

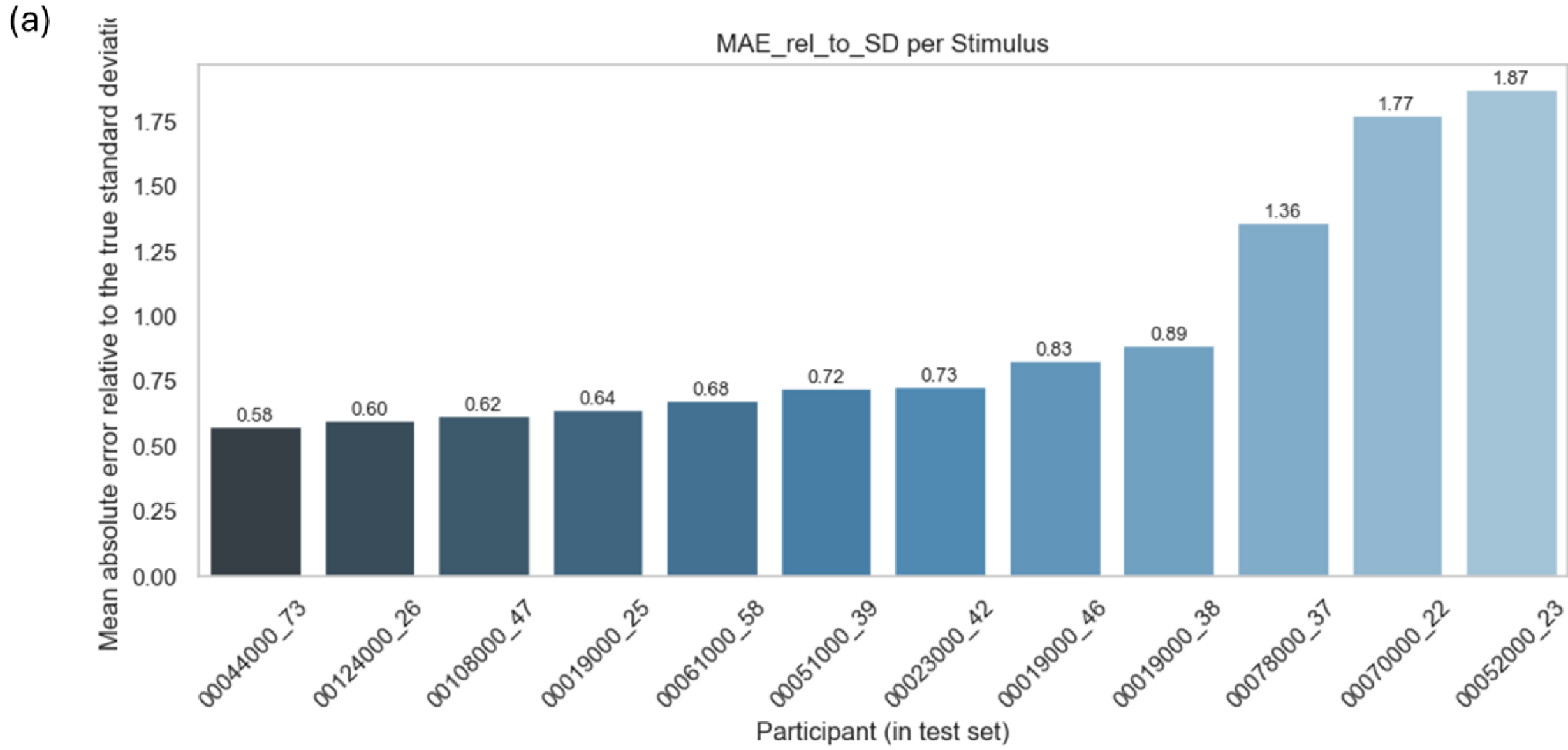

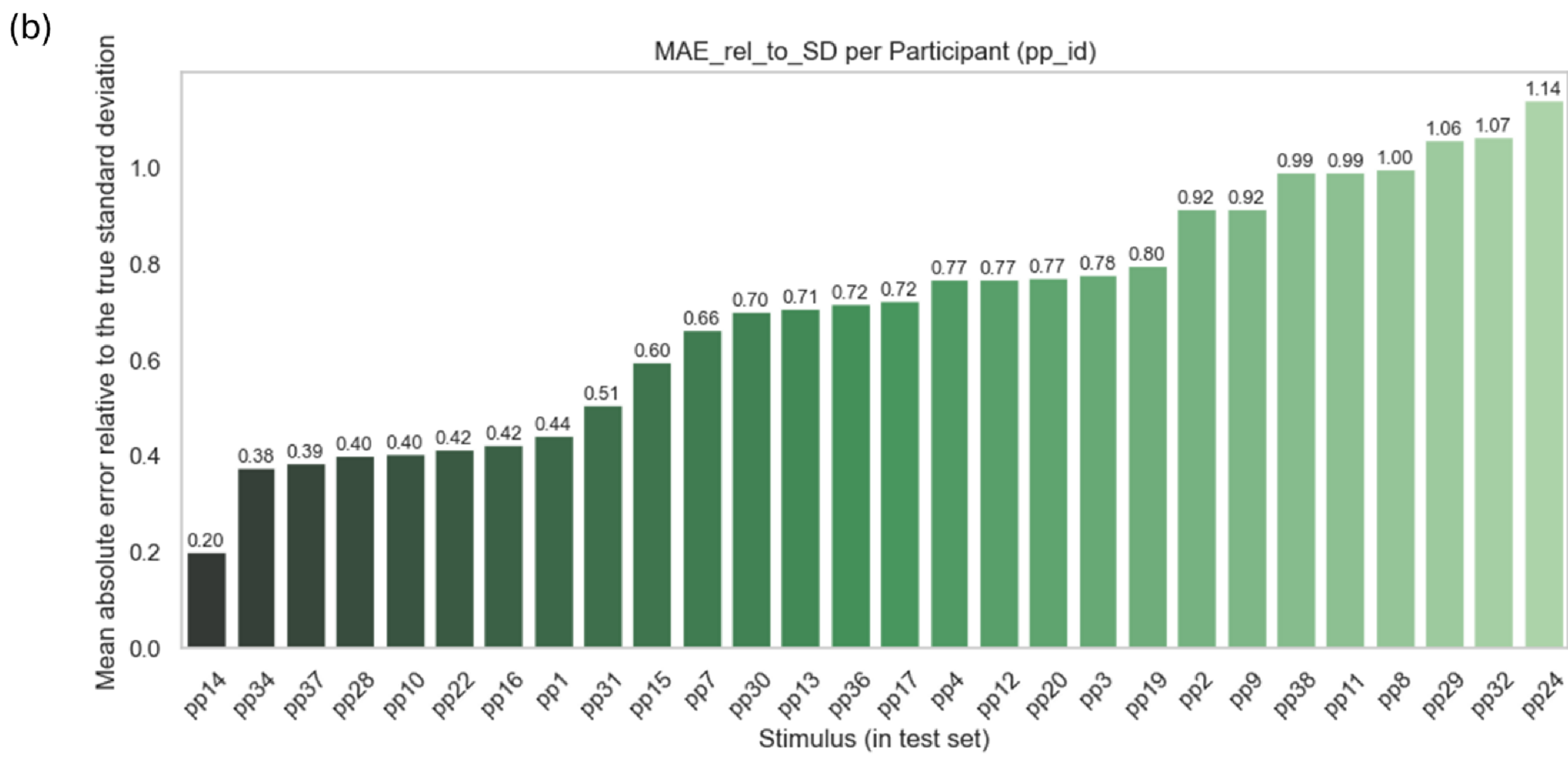

Fig. S11. Associations between regression model performance and scan- or participant-related factors. (a) Regression performance (mean absolute error (MAE) relative to the standard deviation of the true observed values) by scan (n = 12), highlighting variability in prediction accuracy across scans. (b) Regression performance by participant (n = 28), showing individual differences in predictability.

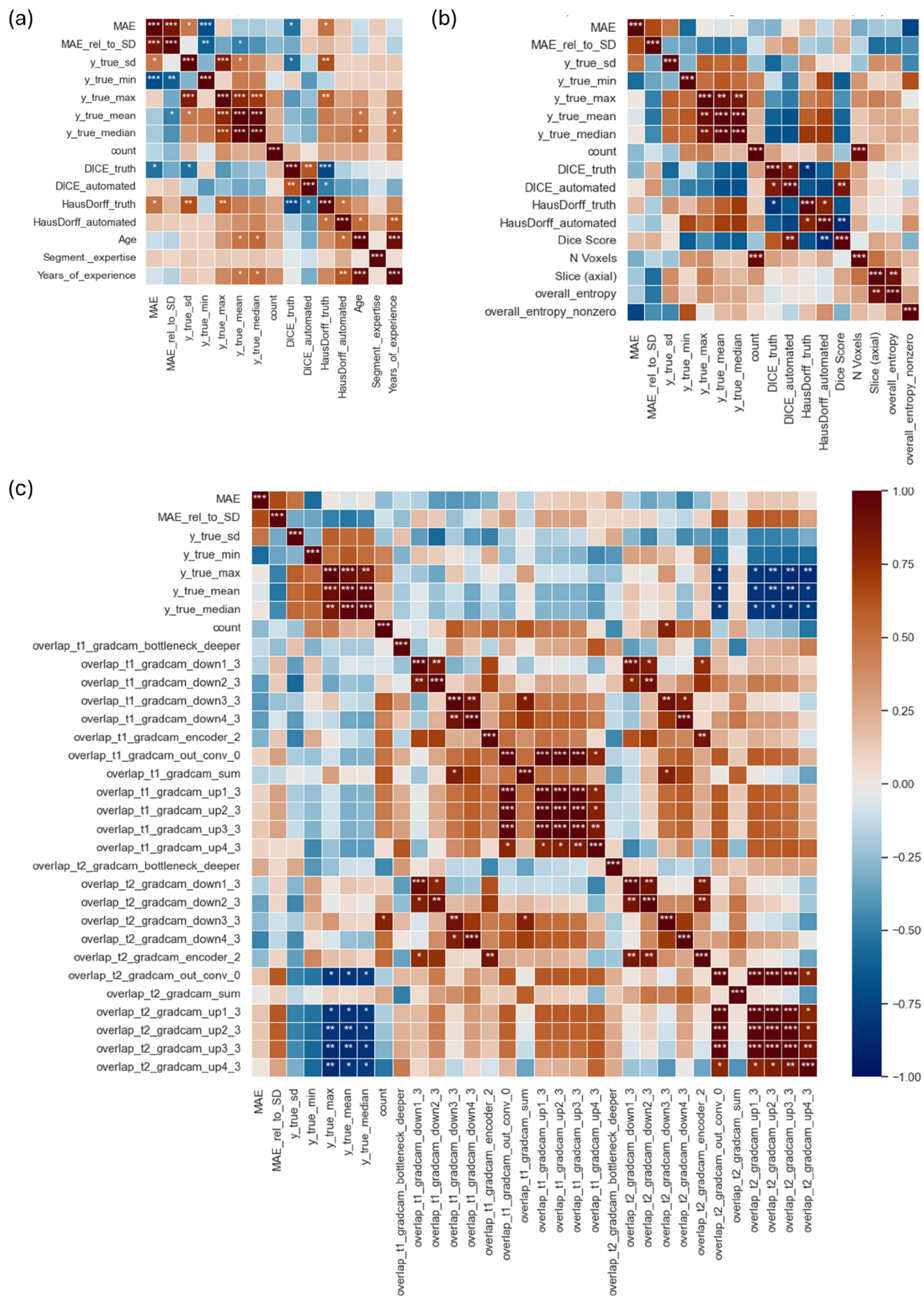

Fig. S12. Associations between regression model performance and scan- or participant-related factors. (a) Participant-level analysis. Correlation between participant-specific variables, such as age and years of experience, and model performance. (b, c) Scan-level analyses. (b) Correlation between scan-related factors,

such as tumor size, axial position, and entropy, and model performance. (c) Correlation between scan-specific measures (e.g., similarity between human and model-derived saliency maps) and regression performance. In all plots, the colors represent the strength of Spearman correlation coefficients between pairs of variables. Statistical significance is indicated by asterisks (*** $p < 0.001$, ** $p < 0.01$, * $p < 0.05$), with p-values corrected for multiple comparisons using the Benjamini–Hochberg False Discovery Rate (FDR) procedure.

Table S1. Summary statistics of the scan-related variables, summarized per experimental group. Values are presented as mean ± standard deviation across trials in each group. Dice score: Measures the overlap between automated and ground truth segmentations (0 = no overlap, 1 = perfect overlap). N Voxels: Indicates tumor volume (number of 1 mm^3 voxels). Slice (axial): Axial slice position (standardized across patients: 1 to 155 - caudal to cranial). Image entropy: Shannon entropy used to quantify the heterogeneity of pixel intensities within the brain (background excluded; 0 = uniform intensity (homogeneous), 8 = maximal heterogeneity or randomness).

Metric	Group 1	Group 2	Group 3	Overall
**Autosegmentation Dice Score**	0.7 ± 0.5	0.8 ± 0.1	0.8 ± 0.2	0.8 ± 0.3
**N Voxels**	1012.5 ± 1286.1	517.3 ± 412.6	547.8 ± 306.2	692.5 ± 761.0
**Slice (axial)**	44.8 ± 20.2	35.3 ± 16.8	39.0 ± 9.8	39.7 ± 15.2
**Image entropy**	7.2 ± 0.2	7.08 ± 0.3	7.2 ± 0.2	7.1 ± 0.2

Table S2. Detailed listing of the MRI scans used in the experiment including group labels, source file (patient_axialposition), automated segmentation performance (Dice Score) and tumor properties including tumor size, axial position, and image entropy. Dice score: Measures overlap between automated and ground truth segmentations (0 = no overlap, 1 = perfect overlap). N Voxels: Indicates tumor size (number of voxels, full scan). Slice (axial): Axial slice position (standardized across patients, 1 = bottom of the brain, 155 = top of the brain). Image entropy: Shannon entropy used to quantify the heterogeneity of pixel intensities within brain tissue only (background excluded); 0 means uniform intensity (homogeneous), higher values indicate greater heterogeneity or randomness.

Group	Source File	Dice Score	Size	N Voxels	Slice (axial)	Entropy
1	00019000_25	0.06	Medium (500-1000)	854	25	7.28
1	00023000_42	0.94	Small (<500)	213	42	7.21
1	00044000_73	0.98	Large (>1000)	2877	73	7.18
1	00051000_39	0.95	Small (<500)	106	39	7.51
2	00019000_38	0.63	Large (>1000)	1061	38	7.37
2	00052000_23	0.93	Medium (500-1000)	584	23	6.72
2	00061000_58	0.84	Small (<500)	102	58	6.93
2	00070000_22	0.92	Small (<500)	322	22	6.88
3	00019000_46	0.51	Medium (500-1000)	659	46	7.20
3	00078000_37	0.82	Medium (500-1000)	855	37	7.40
3	00108000_47	0.91	Small (<500)	130	47	6.89
3	00124000_26	0.86	Medium (500-1000)	547	26	7.14

Table S3. Description of behavioural annotation extracted for each task for each scan.

Variable	Explanation	Quantified across
GT entry time [ms]	Time in ms from the task start to the first fixation within the ground truth tumour area.	Verbal descriptive task (T1) and segmentation task (T2)
GT dwell time [%]	Percentage of total fixation duration within the ground truth tumour area.	Verbal descriptive task (T1) and segmentation task (T2)
GT fixations [%]	Percentage of all fixations (number) within the ground truth tumour area.	Verbal descriptive task (T1) and segmentation task (T2)
Fixation count	Total number of fixations recorded during each task for each scan	Verbal descriptive task (T1) and segmentation task (T2)
Fixation dispersion	Assessing the proportion of image pixels that was explored. Unique pixels with fixation/total pixels.	Verbal descriptive task (T1) and segmentation task (T2)
Fixation entropy	Shannon entropy of the fixation locations. Assessing the randomness of attention patterns.	Verbal descriptive task (T1) and segmentation task (T2)
Fixation duration [avg, ms]	Average fixation duration across the entire task	Verbal descriptive task (T1) and segmentation task (T2)
Dice: own mask vs. attention	The Dice coefficient between the self-drawn tumour segmentation and the Otsu-binarised attention map. Assessing the overlap between areas of high attention and the self-defined tumour area.	Verbal descriptive task (T1) and segmentation task (T2)
Dice: GT mask vs. attention	The Dice coefficient between the BraTS ground truth segmentation mask and the Otsu-binarised attention map. Assessing the overlap between areas of high attention and the true tumour area.	Verbal descriptive task (T1) and segmentation task (T2)

Scan path length [pixels]	The total number of pixels resulting from adding up all distances between consecutive fixations.	Verbal descriptive task (T1) and segmentation task (T2)
Saccade count	Total number of saccades during a task.	Verbal descriptive task (T1) and segmentation task (T2)
Saccade amplitude [pixel]	Average saccade amplitude throughout the task.	Verbal descriptive task (T1) and segmentation task (T2)
Saccade peak velocity [pixel/ms]	Average saccade peak velocity throughout the task.	Verbal descriptive task (T1) and segmentation task (T2)
Saccade velocity [pixel/ms]	Average saccade average velocity throughout the task.	Verbal descriptive task (T1) and segmentation task (T2)
Task duration [ms]	The length of from task start to task end in ms.	Verbal descriptive task (T1) and segmentation task (T2)
Mouse velocity [pixel/ms]	The average velocity of the computer mouse movement in pixel/ms across the task.	Only segmentation task (T2)
Number of loops	Number of iterations of drawing a mouse-drawn tumour segmentation. Considering fully closed loops only.	Only segmentation task (T2)
Dice: GT mask	Dice coefficient of the own mouse-drawn outline against the ground truth segmentation.	Only segmentation task (T2)
Dice: Unet mask	Dice coefficient of the own mouse-drawn outline against the UNET segmentation.	Only segmentation task (T2)

Table S4. Summary statistics of the behavioural metrics summarised per trial (n = 110). The four variables marked with an astericks were not included in the analysis but reported here as a means of assessing the eye-tracker precision. Detailed descriptions of variable names can be found in Table S3 .

Metric	Mean	SD	Min	Max
Number of loops	1.38	0.95	1.00	5.00
Dice: GT mask	0.73	0.20	0.11	0.95
Dice: Unet mask	0.62	0.27	0.00	0.92
Saccade count (T2)	23.35	20.62	3.00	149.00
Fixation count (T2)	25.81	21.04	4.00	154.00
Task duration [ms] (T2)	17556.66	10775.03	4656.00	53359.00
Fixation entropy (T2)	2.99	0.78	0.81	4.96
Saccade amplitude [pixel] (T2)	1.59	0.63	0.53	4.47
Saccade peak velocity [pixel/ms] (T2)	132.16	62.09	72.08	568.03
Saccade velocity [pixel/ms] (T2)	80.03	26.25	50.09	255.33
Mouse velocity [pixel/ms] (T2)	78.53	55.18	14.67	455.05
Fixation duration [avg, ms] (T2)	787.90	532.49	256.29	3671.09
Fixation RMS precision [avg, pixel] (T2)*	0.01	0.00	0.01	0.02
Fixation SD precision [avg, pixel] (T2)*	0.04	0.01	0.02	0.08
Saccade count (T1)	66.38	37.16	10.00	196.00
Fixation count (T1)	79.27	40.48	14.00	196.00
Task duration [ms] (T1)	35505.87	14932.34	7147.00	59984.00
Fixation entropy (T1)	3.50	0.66	2.26	5.18
Saccade amplitude [pixel] (T1)	2.29	0.62	1.15	4.11
Saccade peak velocity [pixel/ms] (T1)	164.32	40.88	85.90	343.63
Saccade velocity [pixel/ms] (T1)	99.11	17.69	63.28	165.71
Fixation duration [avg, ms] (T1)	429.87	182.78	237.02	1635.07
Fixation RMS precision [avg, pixel] (T1)*	0.02	0.00	0.01	0.03
Fixation SD precision [avg, pixel] (T1)*	0.05	0.02	0.02	0.09
GT fixations [%] (T1)	0.39	0.23	0.00	0.92
GT fixations [%] (T2)	0.39	0.21	0.00	0.84
GT dwell time [%] (T1)	0.42	0.23	0.00	0.93
GT dwell time [%] (T2)	0.43	0.25	0.00	0.96
GT entry time [ms] (T1)	895.67	2789.32	0.00	27742.00
GT entry time [ms] (T2)	869.46	2135.03	0.00	13287.00
Dice: own mask vs. attention (T1)	0.42	0.20	0.00	0.82
Dice: GT mask vs. attention (T1)	0.43	0.19	0.00	0.83
Dice: own mask vs. attention (T2)	0.45	0.19	0.04	0.88
Dice: GT mask vs. attention (T2)	0.43	0.18	0.01	0.81

Table S5. Mixed-effects linear regression predicting participant segmentation performance based on automated segmentation performance. A random intercept for each participant accounts for baseline individual variability. The automated Dice score is a significant predictor of participant performance.

**Fixed Effect**	**Estimate**	**Std. Error**	**z-value**	**p-value**	**[95% CI]**
Intercept	0.5	0.1	9.8	<0.001	[0.4, 0.6]
Dice Score	0.3	0.1	4.9	<0.001	[0.2, 0.4]
**Random Effect**					
Group Variance	0.001	0.01			

Table S6. Description of point-level annotation variables extracted around each point (20 pixel radius) on the mouse- based annotation outline.

**Variable**	**Explanation**	**Quantified across**
Mouse velocity [pixel/ms]	Average mouse velocity [pixels/ms] within 20-pixel radius of the outline point; minmax normalised within participant	Segmentation task
Fixation count	Number of fixations within 20-pixel radius of the outline point; minmax normalised within participant	Verbal descriptive task (T1) and segmentation task (T2)
Fixation duration [ms]	Average of fixation duration [ms] of all fixations within the 20-pixel radius around the considered outline point; minmax normalised within participant	Verbal descriptive task (T1) and segmentation task (T2)
Saccade amplitude [pixel]	Average saccade amplitude [pixels] within 20-pixel radius; imputed with nearest saccade if none present; minmax normalised within participant	Verbal descriptive task (T1) and segmentation task (T2)
Saccade peak velocity [pixel/ms]	Average saccade peak velocity [pixels/ms] within 20-pixel radius; imputed with nearest saccade if none present; minmax normalised within participant	Verbal descriptive task (T1) and segmentation task (T2)
Saccade velocity [pixel/ms]	Average saccade average velocity [pixels/ms] within 20-pixel radius; imputed if none present; minmax normalised within participant	Verbal descriptive task (T1) and segmentation task (T2)
Participant attention	Mean value in the normalised fixation map within 20-pixel radius; minmax normalised within participant and task	Verbal descriptive task (T1) and segmentation task (T2)

Attention difference (T1 - T2)	Difference between task-specific fixation map values in 20-pixel radius; unnormalised	Per scan
Entropy	Average image entropy within 20-pixel radius; unnormalised	Per scan
Probability	Mean segmentation probability from the U-Net within a 20-pixel radius; unnormalised	Per scan
GradCAM *layer*	Mean gradCAM saliency across all U-Net layers (encoder, down1, down2, down3, down4, bottleneck, up1, up2, up3, up4, out-convolutional)within 20-pixel radius; min-max normalised into range [0,1]	Per scan
GradCAM sum	Summed saliency across all U-Net layers; min-max normalised into range [0,1]	Per scan
Mean minimal distance to peers (uncertainty) [pixel]	Mean minimal distance [pixels] to other participants' outlines for same scan	Segmentation task only (Task 2)
Distance to ground truth (segmentation error) [pixel]	The minimal distance [pixels] between the point on the self-drawn outline to any point on the ground truth tumour segmentation outline.	Segmentation task only (Task 2)

Table S7 . Summary statistics of the point-level annotation variables (behavioural and image-based) defined in Supplementary Table S6 across all mouse-track outline points (n = 20,238) from all participants and scans. All the image-based variables were left unnormalised. All behavioural variables except for the uncertainty and segmentation error were min-max normalised within each participant. For the regression and predictive analysis, the uncertainty was log-transformed.

**Variable**	**Mean**	**SD**	**Min**	**Max**
Mouse velocity [pixel/ms]	0.36	0.47	0.00	1.00
Fixation count (T1)	0.12	0.18	0.00	1.00
Fixation duration [ms] (T1)	0.10	0.17	0.00	1.00
Saccade amplitude [pixel] (T1)	0.30	0.20	0.00	1.00
Saccade peak velocity [pixel/ms] (T1)	0.26	0.21	0.00	1.00
Saccade velocity [pixel/ms] (T1)	0.29	0.21	0.00	1.00
Fixation count (T2)	0.15	0.20	0.00	1.00
Fixation duration (T2)	0.11	0.19	0.00	1.00
Saccade amplitude [pixel] (T2)	0.22	0.18	0.00	1.00
Saccade peak velocity [pixel/ms] (T2)	0.20	0.19	0.00	1.00
Saccade velocity [pixel/ms] (T2)	0.20	0.19	0.00	1.00
Participant attention (T1)	0.25	0.26	0.00	1.00
Participant attention (T2)	0.30	0.25	0.00	1.00
Attention difference (T1 - T2)	0.47	0.19	0.00	1.00
Entropy	0.80	0.15	0.11	0.97
Probability	0.24	0.27	0.00	1.00
GradCAM encoder	0.33	0.25	0.00	0.95
GradCAM down1	0.21	0.23	0.00	0.84
GradCAM down2	0.19	0.23	0.00	0.86
GradCAM down3	0.32	0.23	0.00	0.90
GradCAM down4	0.22	0.19	0.00	0.89
GradCAM bottleneck	0.33	0.22	0.00	0.98
GradCAM up1	0.09	0.14	0.00	0.85
GradCAM up2	0.10	0.14	0.00	0.83
GradCAM up3	0.12	0.14	0.00	0.77
GradCAM up4	0.21	0.18	0.00	0.87
GradCAM out-convolutional	0.09	0.14	0.00	0.85
GradCAM sum	0.29	0.16	0.01	0.88
Distance to ground truth [pixel]	16.01	22.97	0.50	172.00
Uncertainty (minimal distance to peers) [pixel]	18.97	18.78	1.69	149.65

Table S8. Fixed effects from a linear mixed model. Significance codes: ***p < 0.001, **p <0 .01, *p < 0.05. Abbreviations: orig - original, VIF - variance inflation factor, RF - random forest.

**Predictor**	**Estimate (orig)**	**P**	**Estimate (VIF)**	**P**	**Estimate (RF)**	**P**
(Intercept)	1.3	< .001***	1.31	<.001***	1.30	<.001***
Fixation count (T1)	0.1	.027*	—	—	—	—
Fixation duration (T1)	-0.2	< .001***	-0.12	<.001***	—	—
Saccade amplitude (T1)	-0.0	.049*	-0.02	.059	—	—
Saccade peak velocity (T1)	-0.1	.058·	0.02	.099	—	—
Saccade velocity (T1)	0.1	.026*	—	—	—	—
Fixation count (T2)	0.0	.552	0.01	.508	—	—
Fixation duration (T2)	-0.0	.015*	-0.02	.065	—	—
Saccade amplitude (T2)	-0.1	< .001***	-0.00	.821	—	—
Saccade peak velocity (T2)	-0.3	< .001***	0.01	.297	—	—
Saccade velocity (T2)	0.4	< .001***	—	—	—	—
Participant attention (T1)	-0.1	.066·	0.03	.016*	—	—
Participant attention (T1)	0.1	< .001***	0.06	<.001***	-0.11	<.001***
Attention difference (T1 - T2)	0.1	.015*	0.11	<.001***	—	—
Mouse velocity	0.1	< .001***	0.07	<.001***	0.05	.001**
Entropy	0.0	.038*	—	—	—	—
Probability	0.0	.407	-0.21	<.001***	-0.36	<.001***
GradCAM bottleneck	-0.1	< .001***	-0.39	<.001***	-0.42	<.001***
GradCAM down1	-0.2	< .001***	0.40	<.001***	0.44	<.001***
GradCAM down2	0.5	< .001***	-0.10	<.001***	-0.13	<.001***
GradCAM down3	0.0	.028*	-0.26	<.001***	-0.36	<.001***
GradCAM down4	-0.1	< .001***	0.12	<.001***	0.13	<.001***
GradCAM encoder	0.2	< .001***	—	—	—	—

GradCAM out-convolutional	9.9	< .001***	0.17	<.001***	—	—
GradCAM sum	-1.0	< .001***	0.91	<.001***	—	—
GradCAM up1	-7.8	< .001***	—	—	—	—
GradCAM up2	-0.0	.939	—	—	—	—
GradCAM up3	-1.0	< .001***	-0.80	<.001***	—	—
GradCAM up4	-0.3	< .001***	—	—	—	—

Table S9 . Descriptive statistics for similarity between the average human attention maps across tasks and Grad-CAM saliency attention maps summarized across all scans (n = 12). Similarity is quantified using two distinct metrics: Otsu binarized masks are compared using the Dice coefficient and non-binarized full maps are compared using cosine similarity.

	Dice similarity of binarised attention and saliency masks				Cosine similarity of original continuous attention and saliency maps			
Variable	Mean	Std	Min	Max	Mean	Std	Min	Max
overlap_t1_gradcam_bottleneck	0.59	0.13	0.33	0.71	0.72	0.12	0.46	0.88
overlap_t2_gradcam_bottleneck	0.56	0.13	0.34	0.74	0.71	0.13	0.49	0.88
overlap_t2_gradcam_up4	0.51	0.19	0.13	0.84	0.66	0.18	0.22	0.92
overlap_t1_gradcam_up4	0.50	0.15	0.12	0.73	0.64	0.17	0.20	0.83
overlap_t1_gradcam_up3	0.37	0.16	0.06	0.69	0.52	0.16	0.12	0.73
overlap_t2_gradcam_up3	0.36	0.15	0.06	0.56	0.52	0.16	0.15	0.71
overlap_t1_out_conv	0.35	0.13	0.11	0.63	0.45	0.16	0.06	0.71
overlap_t2_gradcam_sum	0.34	0.14	0.08	0.53	0.58	0.10	0.39	0.76
overlap_t1_gradcam_up2	0.33	0.17	0.03	0.68	0.47	0.16	0.09	0.72
overlap_t1_gradcam_sum	0.33	0.16	0.10	0.71	0.61	0.12	0.45	0.81
overlap_t2_gradcam_up2	0.32	0.14	0.04	0.52	0.47	0.15	0.12	0.65
overlap_t1_gradcam_out_conv	0.32	0.17	0.02	0.67	0.45	0.16	0.06	0.71
overlap_t1_gradcam_up1	0.31	0.17	0.01	0.67	0.44	0.16	0.06	0.71

overlap_t2_gradcam_out_conv	0.30	0.14	0.02	0.51	0.44	0.15	0.08	0.61
overlap_t2_gradcam_up1	0.30	0.14	0.02	0.51	0.44	0.15	0.08	0.61
overlap_t2_gradcam_down3	0.26	0.15	0.07	0.61	0.43	0.14	0.27	0.80
overlap_t1_gradcam_down3	0.25	0.20	0.09	0.73	0.44	0.17	0.24	0.78
overlap_t2_out_conv	0.25	0.17	0.00	0.64	0.44	0.15	0.08	0.61
overlap_t2_gradcam_encoder	0.21	0.17	0.00	0.47	0.35	0.21	0.00	0.61
overlap_t1_gradcam_encoder	0.19	0.15	0.00	0.41	0.38	0.23	0.00	0.70
overlap_t2_gradcam_down2	0.18	0.14	0.02	0.39	0.31	0.19	0.03	0.65
overlap_t2_gradcam_down1	0.17	0.18	0.00	0.48	0.29	0.23	0.00	0.65
overlap_t1_gradcam_down2	0.17	0.12	0.02	0.38	0.33	0.21	0.03	0.68
overlap_t2_gradcam_down4	0.16	0.14	0.00	0.42	0.26	0.16	0.04	0.58
overlap_t1_gradcam_down4	0.15	0.16	0.00	0.47	0.27	0.16	0.08	0.62
overlap_t1_gradcam_down1	0.14	0.15	0.00	0.39	0.31	0.26	0.00	0.65
